# *Para*-Hydroxybenzyl Alcohol Delays the Progression of Neurodegenerative Diseases in Models of *Caenorhabditis elegans* through Activating Multiple Cellular Protective Pathways

**DOI:** 10.1155/2022/8986287

**Published:** 2022-03-31

**Authors:** Yu Liu, Yu-Yang Lu, Lv Huang, Lin Shi, Zhuo-Ya Zheng, Jian-Ning Chen, Yuan Qu, Hai-Ting Xiao, Huai-Rong Luo, Gui-Sheng Wu

**Affiliations:** ^1^Key Laboratory for Aging and Regenerative Medicine, Department of Pharmacology, School of Pharmacy, Southwest Medical University, Luzhou, Sichuan 646000, China; ^2^Department of Pharmacy, Daping Hospital, Army Medical University, Chongqing 400042, China; ^3^Central Nervous System Drug Key Laboratory of Sichuan Province, Luzhou, Sichuan 646000, China; ^4^Key Laboratory of Medical Electrophysiology, Ministry of Education & Medical Electrophysiological Key Laboratory of Sichuan, Institute of Cardiovascular Research, Southwest Medical University, Luzhou, Sichuan 646000, China

## Abstract

The traditional Chinese medicine *Gastrodia elata* (commonly called “Tianma” in Chinese) has been widely used in the treatment of rheumatism, epilepsy, paralysis, headache, and dizziness. Phenolic compounds, such as gastrodin, *para*-hydroxybenzyl alcohol (HBA), *p*-hydroxybenzaldehyde, and vanillin are the main bioactive components isolated from *Gastrodia elata*. These compounds not only are structurally related but also share similar pharmacological activities, such as antioxidative and anti-inflammatory activities, and effects on the treatment of aging-related diseases. Here, we investigated the effect of *para*-hydroxybenzyl alcohol (HBA) on neurodegenerative diseases and aging in models of *Caenorhabditis elegans* (*C. elegans*). Our results showed that HBA effectively delayed the progression of neurodegenerative diseases, such as Alzheimer's disease, Parkinson's disease, and Huntington's disease in models of *C. elegans*. In addition, HBA could increase the average lifespan of N2 worms by more than 25% and significantly improve the age-related physiological functions of worms. Moreover, HBA improved the survival rate of worms under stresses of oxidation, heat, and pathogenic bacteria. Further mechanistic investigation revealed that HBA could activate FOXO/DAF-16 and SKN-1 to regulate antioxidative and xenobiotic metabolism pathway. HBA could also activate HSF-1 to regulate proteostasis maintenance pathway, mitochondrial unfolded stress response, endoplasmic stress response and autophagy pathways. The above results suggest that HBA activated multiple cellular protective pathways to increase stress resistance and protect against aging and aging-related diseases. Overall, our study indicates that HBA is a potential candidate for future development of antiaging pharmaceutical application.

## 1. Introduction

The age-related diseases, such as cancer, cardiovascular disorders, diabetes, and neurodegenerative diseases, have increasingly become the main threats to human health. Aging is considered as the main risk factor for neurodegenerative diseases, such as Alzheimer's disease (AD), Parkinson's disease (PD), and Huntington's disease (HD). Recent studies have shown that drug treatment or gene interference may prolong the lifespan, delay the onset of aging-related diseases, and promote health span [1]. Therefore, there is an urgent need to research in these areas to slow down the aging process and prevent the development of aging-related diseases.

The steamed and dried rhizomes of *Gastrodia elata* Blume (GEB) (commonly called “Tianma” in Chinese) is a traditional Chinese herbal medicine that has long been used in the treatment of rheumatism, epilepsy, paralysis, headache, and dizziness [2, 3]. GEB has antioxidant and free radical scavenging activities [4, 5], could attenuate the inflammatory response in rheumatoid arthritis [6, 7], and protect against neuronal damage by inhibiting the NF-*κ*B and arachidonic acid metabolism pathways [8]. GEE could inhibit NO production and the expression of inducible nitric oxide synthase (iNOS) and cyclooxygenase-2 (COX-2) upon lipopolysaccharide (LPS) stimulation in RAW264.7 macrophages [9]. The anti-inflammatory and antioxidative activities of GEB accounted for the cure effects of many disorders, such as neurological disorders [10–12] and aging-related disorders [13–15]. GEB could protect against glutamate-induced apoptosis in IMR-32 human neuroblastoma cells [16], cerebral ischemia [17], corticosterone-induced apoptosis in PC12 cells [18], and scopolamine-induced learning and memory deficits in rats [19]. The neuroprotective effects of GE might be also mediated through the A(2A)-R/cAMP/PKA/CREB-dependent pathway [20, 21], such as to attenuate mutant Huntingtin aggregation [22], reduce amyloid beta-peptide-induced neuronal cell death in vitro [23], ameliorate circadian rhythm disorder-induced mice memory impairment [24], and improve epilepsy [25]. GEB could ameliorate depression by regulating monoamine and neurotrophic pathways [26–29]. GEB could also improve spatial memory and the expression of choline acetyltransferase in animal models of AD [30]. Water extracts of GEB, gastrodin, and 4-HBA could prevent the locomotion defects and the neuronal loss via glial Nrf2/Mad signaling in PD models of flies and mice [31]. GEB could also ameliorate the impairment of cholesterol and glucose metabolism and blood flow by improving hepatic insulin signaling in ORX rats [32].

Among the over 81 compounds identified in GEB, the main bioactive constituents are polysaccharides and phenolic compounds, such as phenolic glycoside gastrodin, cholesterol, *p*-hydroxyl benzyl alcohol, *p*-hydroxybenzaldehyde, and vanillin [10]. Polysaccharides have a potential protective effect against chemotherapy-induced and vincristine-evoked neuropathic pain via the inhibition of neuroinflammation [18, 33]. The phenolic compounds isolated from GEB not only are structurally related but also have similar pharmacological activities, such as anti-inflammation [5, 34–36] and antioxidative [5, 37], and similar treatment effects on neurological disorders [38–40] and circulatory disorders [41]. The HBA oxidation product 4-hydroxybenzoic acid could increase longevity and stress resistance in *C. elegans* through antioxidant activity and DAF-16/FOXO activation [42]. The antioxidative and anti-inflammatory activities [43, 44] of HBA could accelerate wound healing [45], improve memory [46–49], and protect against neuroexcitatory toxicity [50, 51] and nonalcoholic fatty liver disease (NAFLD) [52]. In the screening of mutated *E. coli* strains against neurodegenerative diseases in models of *C. elegans*, we found that one strain containing high content of HBA has the relatively higher activity against neurodegenerative diseases (unpublished data). So, we are wondering whether HBA has antiaging activity, and if so, what are the mechanisms?

*C. elegans* has been widely used in the research on aging and age-related diseases for its highly conserved molecular pathways, clear genetic background, short lifespan, and transparent body [53]. In this study, we used the *C. elegans* models to explore the effect and mechanism of HBA against neurodegenerative diseases and various stress resistances.

## 2. Materials and Methods

### 2.1. Strains and Chemicals

Unless otherwise indicated, all strains were obtained from the *Caenorhabditis* Genetics Center (CGC) and kept at the appropriate temperature as previously described [54]. The strains used in this study are as follows: the wild-type strain Bristol N2 (Bristol, wild-type), DA1116 *eat-2(ad1116) II*, VC199 *sir-2.1(ok434) IV*, MQ887 *isp-1(qm150) IV*, CB4876 *clk-1(e2519) III*, CB1370 *daf-2(e1370) III*, CF1038 *daf-16(mu86) I*, RB759 *akt-1(ok525) V*, VC204 *akt-2(ok393) X*, RB754 *aak-2(ok524) X*, EU1 *skn-1(zu67) IV*, PS3551 *hsf-1(sy441) I*, RB1206 *rsks-1(ok1255) III*, CF1553 *muIs84 [(pAD76) sod-3p::GFP + rol-6(su1006)]*, CL2166 *dvIs19 [(pAF15)gst-4p::GFP::NLS] III*, SJ4100 *zcIs13 [hsp-6p::GFP + lin-15(+)]*, SJ4005 *zcIs4 [hsp-4::GFP] V*, SJ4058 *zcIs9 [hsp-60::GFP + lin-15(+)]*, CL4176 *dvIs27 [myo-3p::A-Beta (1-42)::let-851 3*′*UTR) + rol-6(su1006)] X,* LD1 *ldIs7 [skn-1b/c::GFP + rol-6(su1006)]*, CL2006 *dvIs2 [pCL12(unc-54/human Abeta peptide 1-42 minigene) + rol-6(su1006)]*, CL2122 *dvIs15 [(pPD30.38) unc-54(vector) + (pCL26) mtl-2::GFP]*, CL2355 *dvIs50 [pCL45 (snb-1::Abeta 1-42::3*′*UTR(long) + mtl-2::GFP]*, NL5901 *pkIs2386 [unc-54p::alpha-synuclein::YFP + unc-119(+)]*, AM141 *rmIs133 [unc-54p::Q40::YFP]*, BC12921 *sIs10729 [rCes T12G3.1::GFP + pCeh361]*, and BZ555 *egIs1 [dat-1p::GFP]*. All strains were grown and maintained on NGM plates inoculated with *E. coli* OP50. HBA (4-hydroxybenzyl alcohol) was purchased from Shanghai Standard Technology Co. Ltd. and dissolved in ddH_2_O. NGM plates containing HBA were equilibrated overnight before use.

### 2.2. Paralysis Assay

Different concentrations of HBA were used to treat *C. elegans*, and the optimal concentration was determined according to its anti-AD activity. The temperature-sensitive transgenic strain (CL4176) was used as the AD model and kept at 16°C on NGM plates containing HBA (0 *μ*M, 50 *μ*M, 100 *μ*M, 200 *μ*M, or 400 *μ*M). L3 stage larvae were induced to express human A*β*_1-42_ by increasing the incubation temperature from 16°C to 25°C for 34 hours. Then, worms were scored for paralysis every 2 hours. The L3 larvae of transgenic strain CL2006 were transferred to NGM plates containing different concentrations of HBA (0 *μ*M, 50 *μ*M, 100 *μ*M, 200 *μ*M, or 400 *μ*M), and then, the paralysis of worms was monitored every day. A worm is considered paralyzed if it cannot move its body when touched or it could only move its head when eating or produce a “halo” resulting from clearing the bacteria [55]. The assays are repeated at least three times. Prism 6.0 was used for statistical analysis, and the log-rank test was used to calculate the *p* value.

### 2.3. Lifespan Assay

All experiments were performed under the optimal concentration of HBA determined by paralysis assay. The synchronized *C. elegans* young adults were transferred to NGM plates containing 200 *μ*M of HBA and 50 *μ*M of FUDR (to prevent egg laying). The day of transfer was designated as day 0. Then, the worms were transferred to fresh plates every other day until all died. The living status of the worms was monitored every day. The abnormal worms were eliminated. GraphPad software was used to calculate the Kaplan–Meier lifetime and *p* value. All assays were repeated independently for three times.

### 2.4. Health Status Assay

Eight experimental groups of worms in four NGM plates coated with 200 *μ*M of HBA and in four control plates were used in each health status assay. About every 100 individuals of L4 larvae (or young adults) either from wild-type Bristol N2 or transgenic strain CL2006 were transferred to each plate. Then, worms were separated into two big groups. Each group contains N2 worms and CL2006 worms in the control plate and plate with HBA, respectively. Then, the two big groups of worms were cultured either for 3 or 7 days. After that, N2 and CL2006 worms were transferred to fresh OP50 plates and kept at 20°C until the next day for phenotypic assays.

For body bending measurement [56], every group of three worms was gently transferred to a plate with a drop of M9 buffer and left standing at room temperature for 20 s. Then, the appearance of the entire body curvature was monitored under a microscope and counted for 20 s. The curvature refers to the maximum curvature of the worm in the form of a sine wave from one end to the opposite direction. Reverse bending in the same direction is not included in the count. At least three independent repeated experiments were performed.

For lipofuscin accumulation assay, the worms were anesthetized with sodium azide (2%). Fluorescence pictures were collected with a fluorescence microscope (Leica DM6B) on the 8th day of adults to determine the intestinal lipofuscin levels. ImageJ software was used to quantify the fluorescence intensity by determining the average pixel intensity in the intestine of each worm. At least three independently repeated experiments were performed.

Chemotaxis assays were performed as described in the previous report [57]. The synchronized larvae of transgenic strain CL2355 expressing A*β*_1-42_ treated with HBA and its control strain CL2122 without HBA treatment were incubated at 16°C for 36 hours and then at 23°C for another 36 hours. During assay, about 80 individuals from either strain were placed at the center of the assay plate (100 mm) containing an “attractant” spot and a control spot. The “attractant” spot contains 1 *μ*L of sodium azide solution (0.25 M) and 1 *μ*L of odorant (0.1% benzaldehyde in 100% ethanol). The control spot contains 1 *μ*L of control odorant (100% ethanol) and 1 *μ*L of sodium azide solution. The assay plate was incubated at 23°C for one hour. Then, the number of worms in each quadrant was counted. The following formula was used to calculate the chemotactic index (CI): CI = (the number of worms in the lure quadrant − the number of worms in the control quadrant)/total number of worms. At least three independently repeated experiments were performed.

### 2.5. Stress Resistance Assay

For oxidative stress assay, young adults of wild-type N2 and CL2006 worms expressing A*β*_1-42_ were transferred to NGM plates containing 20 mM of paraquat (Sigma) and cultured at 20°C [58]. The worms are monitored every day. If the worms do not respond to the soft touch of the platinum wire pick, they are scored as dead. Kaplan–Meier was performed to analyze the lifespan of the worms, and the log-rank test was used to calculate the *p* value. The assay was independently repeated three times.

For thermotolerance assay, young adults of N2 worms and CL2006 worms expressing A*β*_1-42_ were transferred to fresh plates with or without 200 *μ*M of HBA and incubated at 35°C. Worms were monitored every two hours and recorded as dead when they did not respond to the light touch of the platinum wire pick [58]. Kaplan–Meier life analysis was performed, and the log-rank test was used to calculate the *p* value. The assay was independently repeated three times.

For pathogenic resistance assay, the plates were inoculated with live *Pseudomonas aeruginosa* (PA14) and cultured overnight before use. The worms N2 and CL2006 were transferred to NGM plates with live PA14, incubated at 20°C, monitored daily, and recorded as dead when they did not respond to the gentle touch of the platinum wire pick [58]. Kaplan–Meier was performed to analyze the lifespan of the worms, and the log-rank test was used to calculate the *p* value. The assay was independently repeated three times.

### 2.6. Fluorescence Intensity Analysis

The transgenic strains SJ4005 *zcIs4 [hsp-4::GFP] V*, SJ4100 *zcIs13 [hsp-6p::GFP + lin-15(+)]*, SJ4058 *zcIs9 [hsp-60::GFP + lin-15(+)]*, CF1553 *muIs84 [(pAD76) sod-3p::GFP + rol-6(su1006)]*, CL2166 *dvIs19 [(pAF15)gst-4p::GFP::NLS] III*, LD1 *ldIs7 [skn-1b/c::GFP + rol-6(su1006)]*, and BC12921 *sIs10729 [rCes T12G3.1::GFP + pCeh361]* express GFP-conjugated proteins HSP-4, HSP-6, HSP-60, SOD-3, GST-4, SKN-1, and BEC-1, respectively. The distribution and intensity of the fluorescence in these strains were measured. Approximately, every 100 L4 larvae of these transgenic strains were transferred to an NGM plate with or without 200 *μ*M of HBA and kept for 3 days at 20°C. Then, the photo with green fluorescence of these worms was taken by using a fluorescence microscope (Leica DM6B). The images of at least 30 worms per group were taken. The ImageJ was used to quantify the fluorescence intensity. The assay was repeated at least three times independently.

### 2.7. Reactive Oxygen Species (ROS) Assay

About every 150 synchronized young adult individuals were picked up into each centrifuge tube containing 1 mL of M9 buffer. Then, the tubes were centrifuged at 4200 r/min for 2 minutes. The supernatant was discarded. The worms were washed three times. Subsequently, 998 *μ*L of PBS and 2 *μ*L of 10 mM CM-H_2_DCFDA dye solution were added to the remaining pellet per tube. After that, the tubes were incubated at 35°C for 2 hours. Next, the tubes were centrifuged at 4200 r/min for 2 minutes. The supernatant was discarded. Subsequently, the worms were washed twice with M9 buffer. Then, at least 30 worms from each tube were randomly selected and pictured by using the fluorescence microscope (Leica DM6B) with excitation light at 488 nm and the fluorescence emission at 525 nm [59]. Each experiment was repeated three times.

### 2.8. SOD Activity Detection

Synchronized larvae of strain CL4176 (*n* = 60) were spread on the experimental NGM plate and incubated at 16°C (permissible temperature) for 48 hours. To induce progressive paralysis caused by amyloid, the worms were shifted from 16°C to 25°C and cultured for 36 hours. Then, the worms were collected and resuspended in a homogenization buffer (10 mM Tris-HCl, 150 mM NaCl, and 0.1 mM EDTA, at pH 7.5) and grounded on ice. The decolorization of formazan by the enzymatic reaction of xanthine and xanthine oxidase was analyzed, and the activity of superoxide dismutase (SOD) was measured with a microplate reader. The total superoxide dismutase (T-SOD) assay kit (hydroxylamine method) (Solarbio) and total protein assay kit (with standard: BCA method) (Solarbio) were used to determine protein concentration. The SOD activity was calculated according to protein concentration.

### 2.9. Neurodegenerative Disease-Associated Protein Detection

The A*β* aggregation assay was performed as previously reported [60]. In brief, the synchronized transgenic CL2006 worms treated with or without HBA were incubated until becoming L4 larvae or young adults at 20°C. Then, these worms were collected and fixed at 4°C overnight in 4% of paraformaldehyde/phosphate-buffered saline (PBS). Next, the fixed worms were permeabilized in sample lysis buffer (1% Triton X-100, 5% fresh *β*-mercaptoethanol, 125 mM Tris, pH 7.4) under incubation for 24 hours at 37°C. After that, the worms were stained with 0.125% of thioflavin S (Sigma), dissolved in 50% of ethanol for 2 minutes. Then, the samples were washed and decolorized with ethanol continuously and mounted on a glass slide for microscopic examination and observed under a microscope equipped with a digital camera (DM6B; Leica, Germany). At least 30 individuals were used each time. The experiment was repeated 3 times. Two-tailed Student's *t-*test was used to calculate the *p* value.

In poly-Q polymerization analysis, pictures of the synchronized transgenic worms AM141 *rmIs133 [unc-54p::Q40::YFP]* with and without HBA treatment were taken using the fluorescence microscope (Leica DM6B) on the 3rd and 7th day of adulthood. Then, the poly-Q aggregations were counted in each animal. For each experiment, more than 30 worms at the corresponding stage were used. The experiment was performed independently 3 times.

In *α*-synuclein aggregation analysis, pictures of synchronized transgenic worms NL5901 *(pkIs2386) [unc-54p::alpha synuclein::YFP + unc-119(+)]* with and without HBA treatment were taken by using the fluorescence microscope (Leica DM6B) at the 3rd or 7th day of adulthood. Then, the fluorescence intensity in pictures was quantified to evaluate the *α*-synuclein aggregates in each animal. For each experiment, 30 worms at the corresponding stage were used and the experiment was performed 3 times. The *p* value was calculated by using the *t*-test.

The transgenic strain BZ555 *egIs1 [dat-1p::GFP]* specifically expresses GFP in dopaminergic neurons, which can be injured by 6-OHDA [61]. To induce the selective degeneration of DA neurons, the L3 stage larvae of strain BZ555 were transferred to the NGM plates containing 50 *μ*M of 6-OHDA and 10 mM of ascorbic acid and incubated for 1 h at 20°C. In the meantime, the plates were gently shaken once every 10 minutes. Then, the plates were washed 3 times with M9 buffer. The worms were collected and cultured in an NGM plate containing 200 *μ*M of HBA for 72 h at 20°C [62]. Subsequently, a fluorescence microscope (DM6B; Leica, Germany) was used to take fluorescence pictures of head neurons. The image processing software ImageJ was used for analysis of fluorescence intensity. At least 30 individuals were included in each group. The experiment was repeated independently at least 3 times.

### 2.10. Quantitative RT-PCR Analysis of Gene Expression

Synchronized young adult worms were transferred to six NGM plates (9 cm in diameter) with or without 200 *μ*M of HBA and cultured at 20°C. The total RNA was extracted with using TRIzol A^+^ (Tiangen, China) and converted into cDNA by using a High-Capacity cDNA Reverse Transcription Kit (Applied Biosystems). The expressed genes were amplified and quantified in SYBR Green PCR Mix with the ABI 7500 DNA Analyzer. The relative expression levels of genes were calculated by using the 2^−ΔΔ*CT*^ method and normalized to the expression of gene *cdc-42*. All the primers used in this research are listed in Table S12.

### 2.11. Statistical Analysis

GraphPad Prism 6.0 was used for statistical analysis. For longevity and paralysis determination, Kaplan-Meier survival analysis was performed and the log-rank test was used to calculate the *p* value. For comparison between two groups, the *t*-test was used. One-way analysis of variance (ANOVA) and *t*-test were used for comparison between multiple groups. In our study, 2-tailed Student's *t*-test was used to calculate the *p* value unless otherwise stated and *p* < 0.05 is considered statistically significant.

## 3. Results

### 3.1. HBA Can Delay the Progress of Neurodegenerative Diseases of *C. elegans*

Neurodegenerative diseases are a group of aging-related diseases, such as Parkinson's disease (PD), Alzheimer's disease (AD), and Huntington's disease (HD) [63, 64]. Although each of these diseases has distinct pathogenesis, they are all results from the chronic toxicity of misfolded proteins accumulated with aging [65]. Here, we investigated if HBA has an effect on these diseases in *C. elegans* models.

**Figure 1:**
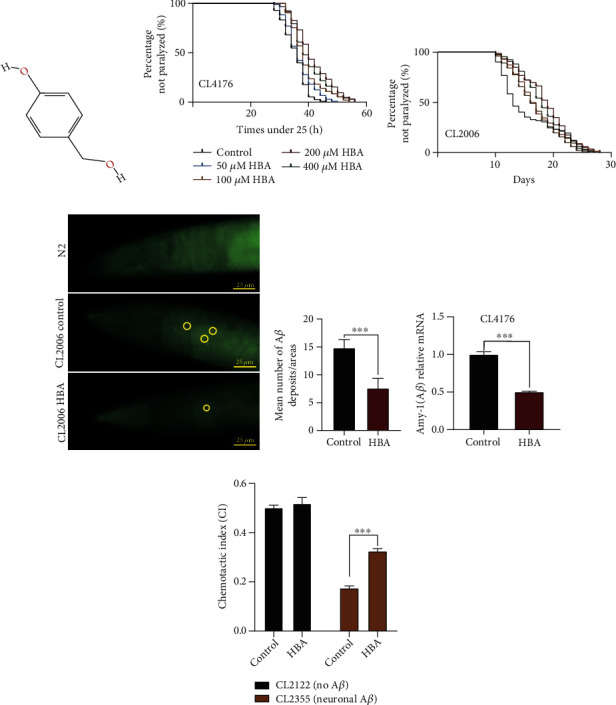
HBA delays the progression of Alzheimer's disease (AD) in models of *C. elegans*. (a) The chemical structure of HBA. (b) Paralysis analysis of *C. elegans* CL4176 *dvIs27 [myo-3p::A-Beta (1-42)::let-851 3*′*UTR) + rol-6(su1006)] X* under ddH_2_O (control) and HBA (50 *μ*M, 100 *μ*M, 200 *μ*M, and 400 *μ*M). (c) Paralysis analysis of strain CL2006 *dvIs2 [pCL12(unc-54/human Abeta peptide 1-42 minigene) + rol-6(su1006)]* under ddH_2_O (control) and HBA (50 *μ*M, 100 *μ*M, 200 *μ*M, and 400 *μ*M). (d) Thioflavin S straining of A*β*_1-42_ in wild-type N2 and transgenic strain CL2006 *(dvIs2)* fed with or without 200 *μ*M HBA. Scale bar, 25 *μ*m. (e) The quantification of A*β* protein aggregation spots indicated by thioflavin S staining in the head of worms CL2006 (*p* < 0.001). (f) Gene expression of A*β*_1-42_ in *C. elegans* CL4176 fed with or without 200 *μ*M of HBA (mean ± SD, *n* = 3). (g) The chemotactic index (CI) of strain CL2355 and the transgenic control strain CL2122 fed with vehicle or 200 *μ*M of HBA. Data were obtained from three repeated experiments with 80 worms in each group. Prism 6.0 was used for statistical analysis, and a *t*-test or log-rank test was used to express statistical significance in *p* value (^∗^*p* < 0.05, ^∗∗^*p* < 0.01, and ^∗∗∗^*p* < 0.001). Statistical details and repeats of these assays are summarized in Tables S1, S2, S5, and S11 (supplementary information).

It is currently recognized that the incidence of AD was caused by the aggregation of toxic amyloid-peptide A*β* protein, which leads to the decline of learning and memory ability [66]. The AD model of transgenic strain CL4176 could be induced to express human A*β* (1-42) when upshifted to the nonpermissive temperature of 23°C. The toxicity of A*β* leads to oxidative stress and finally paralysis of worms. To investigate the anti-AD activity of HBA in *C. elegans*, the onset of paralysis of worms treated with different concentrations of HBA was determined (Figures 1(a) and 1(b)). We found that 200 *μ*M of HBA could significantly delay the onset of paralysis of CL4176 worms by up to 21% (Supplementary Table 1). To investigate whether the alleviation of *C. elegans* paralysis after HBA treatment is related to the A*β* content, we detected the A*β* mRNA levels in CL4176. Our results showed that HBA significantly decreased the mRNA levels of A*β* in CL4176 (Figure 1(f)). Our results showed that HBA could also significantly delay the paralysis of strain CL2006 (which expresses A*β* constitutively) by up to 19% (Figure 1(c)). Additionally, we performed thioflavin S staining on the 7th day of CL2006 after feeding with HBA to measure the effect of HBA on punctate aggregation of A*β*. Our results showed that HBA treatment significantly reduced the A*β* aggregation in CL2006 worms (Figures 1(d) and 1(e)). The perception behavior of *C. elegans* could be a learning indicator for the progression of AD [57]. Chemotaxis experiments showed that HBA significantly ameliorated the neuronal chemotaxis defects in CL2355 worms induced by A*β* (Figure 1(g)).

**Figure 2:**
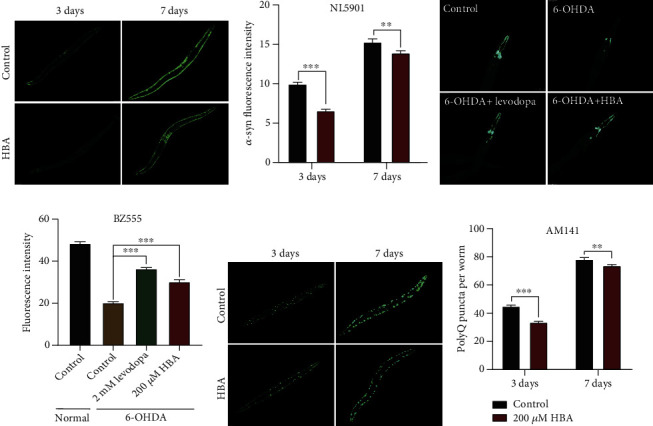
HBA delays the progression of Parkinson's disease (PD) and Huntington's disease (HD) in models of *C. elegans*. (a) The fluorescence picture of *α*-synuclein conjugated with yellow fluorescent protein in *C. elegans* NL5901 *pkIs2386 [unc-54p::alpha-synuclein::YFP + unc-119(+)]*, fed with or without 200 *μ*M of HBA on the 3rd and 7th day of adulthood. (b) The quantification of *α*-synuclein protein aggregation in strain NL5901 (*p* < 0.001). (c) The fluorescence picture of dopaminergic neurons in *C. elegans* strain BZ555 fed with or without 200 *μ*M of HBA after being treated with 6-hydroxydopamine. (d) The quantification of the fluorescence intensity of dopaminergic neurons in the *C. elegans* strain BZ555 *egIs1 [dat-1p::GFP]* (*p* < 0.001). (e) The fluorescence picture of poly-Q conjugated with yellow fluorescent protein in strain AM141 *rmIs133 [unc-54p::Q40::YFP]* fed with or without 200 *μ*M of HBA. (f) The quantification of the aggregation of poly-Q protein in the strain AM141. Prism 6.0 was used for statistical analysis, and the values were expressed as mean ± SEM. The *t*-test or log-rank test were used to express statistical significance in *p* value (^∗^*p* < 0.05, ^∗∗^*p* < 0.01, and ^∗∗∗^*p* < 0.001). Statistical details and repeats of these assays are summarized in Table S2 (supplementary information).

Parkinson's disease (PD) is characteristic of the accumulation of *α*-synuclein in Lewy bodies and loss of dopaminergic neurons in the substantia nigra. The transgenic strains NL5901 *([unc-54p::α-synuclein::YFP + unc-119(+)])* and BZ555 *egIs1 [dat-1p::GFP]* were widely used as PD models for mechanistic research and pharmacological screening. Here, we treated NL5901 worms with HBA and measured the fluorescence intensity and punctate aggregation. We found that on the 3rd and 7th day of HBA treatment, the age-related *α*-syn aggregation was significantly reduced (Figures 2(a) and 2(b)). In the transgenic BZ555 worms, the morphological pattern of dopamine neurons could be observed through the dopamine transporter protein DAT-1::GFP. Selective degeneration of dopamine neurons in *C. elegans* can be induced by 6-hydroxydopamine (6-OHDA) [67]. Our results showed that 6-OHDA decreased the average fluorescence intensity of dopamine neurons in the BZ555 worms from 48.418 to 20.168, while HBA treatment remained the average fluorescence intensity at 30.151 (Figures 2(c) and 2(d)), suggesting that HBA treatment protected the degeneration of head neurons in BZ555 worms.

The worms AM141 (*rmIs133) [unc-54p::Q40::YFP]* expressing polyglutamine fused with yellow fluorescent protein (YFP) were used as the HD model [68]. We found that HBA treated for 3 and 7 days could significantly reduce the polyglutamine aggregation (Figures 2(e) and 2(f)). The above results indicate that HBA could effectively delay the progression of a variety of neurodegenerative diseases in models of *C. elegans*.

### 3.2. HBA Could Increase the Healthy Lifespan of *C. elegans* via Transcription Factor FOXO/DAF-16

**Figure 3:**
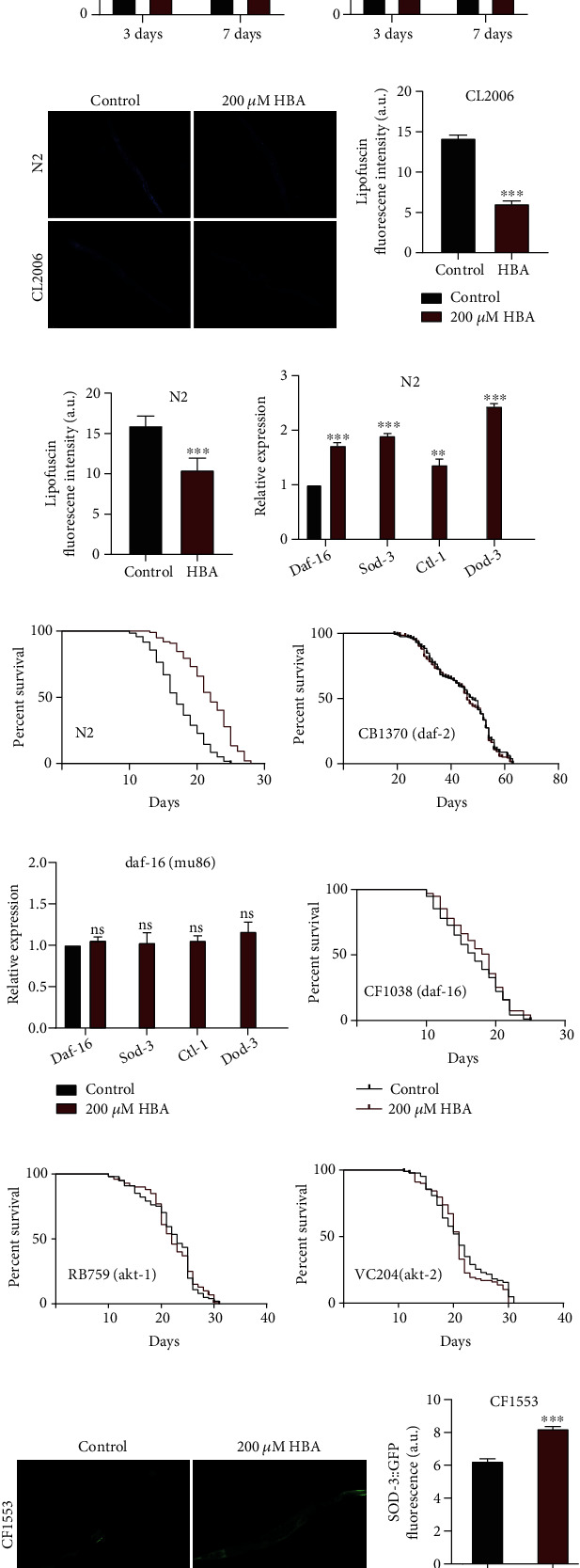
HBA reduces the accumulation of ROS in *C. elegans*. The body bending behavior (body bending times every 20 seconds) on the 3rd and 7th day of adulthood of wild-type (a) N2 and (b) CL2006 fed with 200 *μ*M of HBA (*p* < 0.001). (c) The pictures of lipofuscin deposition on the 7th day of adulthood of wild-type N2 and CL2006 fed with 200 *μ*M of HBA. The quantification of lipofuscin on the 7th day of adulthood of wild-type (d) N2 and (e) CL2006 by using ImageJ. Values are expressed as mean ± SEM. (*p* < 0.001). The mRNA levels of genes in wild-type (f) N2 and (i) null mutant *daf-16 (mu86)* fed with HBA (mean ± SD, *n* = 3). Lifespan analysis of (g) N2, (h) null mutant *daf-2(e1370)*, (j) null mutant *daf-16 (mu86)*, (k) null mutant *akt-1(ok525) V*, and (l) null mutant *akt-2(ok393)*, after being fed with HBA. The (m) fluorescent picture and (n) quantification of CF1553 *sod-3::GFP* expressing SOD-3 conjugated with GFP fed with HBA. Prism 6.0 was used for statistical analysis, and the values were expressed as mean ± SEM. Use the *t*-test or log-rank test to express statistical significance in *p* value (^∗^*p* < 0.05, ^∗∗^*p* < 0.01, and ^∗∗∗^*p* < 0.001). Statistical details and repeats of these assays are summarized in Tables S3, S4, S9, S10, and S11 (supplementary information).

During aging, systemic muscle cells gradually lose their vitality and diminish their activity [69]. To understand whether the HBA could increase their health span, we evaluated the effect of HBA on age-related physiological function, such as body bending in N2 and CL2006 worms. The results showed that HBA significantly enhanced the body bending of N2 (Figure 3(a)) and CL2006 worms (Figure 3(b)). The endogenous intestinal autofluorescence lipofuscin level would accumulate during *C. elegans* aging [69]. Our results showed that HBA significantly decreased the fluorescence intensity of intestinal lipofuscin in N2 and transgenic CL2006 by 34.3% and 54.3% compared with the control group, respectively (Figures 3(c)–3(e)). Furthermore, we found that the 200 *μ*M of HBA could extend the lifespan of wild-type N2 by 25% (Figure 3(g)).

DAF-16 is the homologue of mammalian forkhead transcription factor (FOXO) and is a key regulator for development, metabolism, stress response, and aging [68]. To study whether the lifespan extension induced by HBA depends on DAF-16, we analyzed the lifespan of *daf-16* null mutant *daf-16* (*mu86*) treated with HBA. Our results showed that HBA could not increase the longevity of *daf-16* null mutant worms (Figure 3(j)). In addition, HBA increased the mRNA levels of *daf-16* and its downstream genes (*sod-3*, *ctl-1*, and *dod-3*) in wild-type N2, but not in the null mutants of daf-16 (Figures 3(f) and 3(i)). Consistent with this, the fluorescence of SOD-3::GFP also increased in *C. elegans* CF1553 expressing the fluorescent protein SOD-3::GFP (Figures 3(m) and 3(n)). In *C. elegans*, insulin-like ligands act on insulin receptor DAF-2, which subsequently activate phosphoinositide-3 kinase (PI3K) and regulate the activity of AKT-1 and AKT-2 kinases through phosphorylation of PDK-1. Then, the activities of multiple downstream targets, including the DAF-16/FOXO transcription factor, were regulated. Therefore, we investigated the effect of HBA on the long-live mutants *daf-2(e1370)*, *akt-1* (*ok525*), and *akt-2* (*ok393*). The results showed that HBA could not further extend the lifespan of these long-lived mutants (Figures 3(h) and 3(j)–3(l)).

### 3.3. HBA Could Enhance the Stress Resistance of *C. elegans* by Activating Cellular Protection Pathways

**Figure 4:**
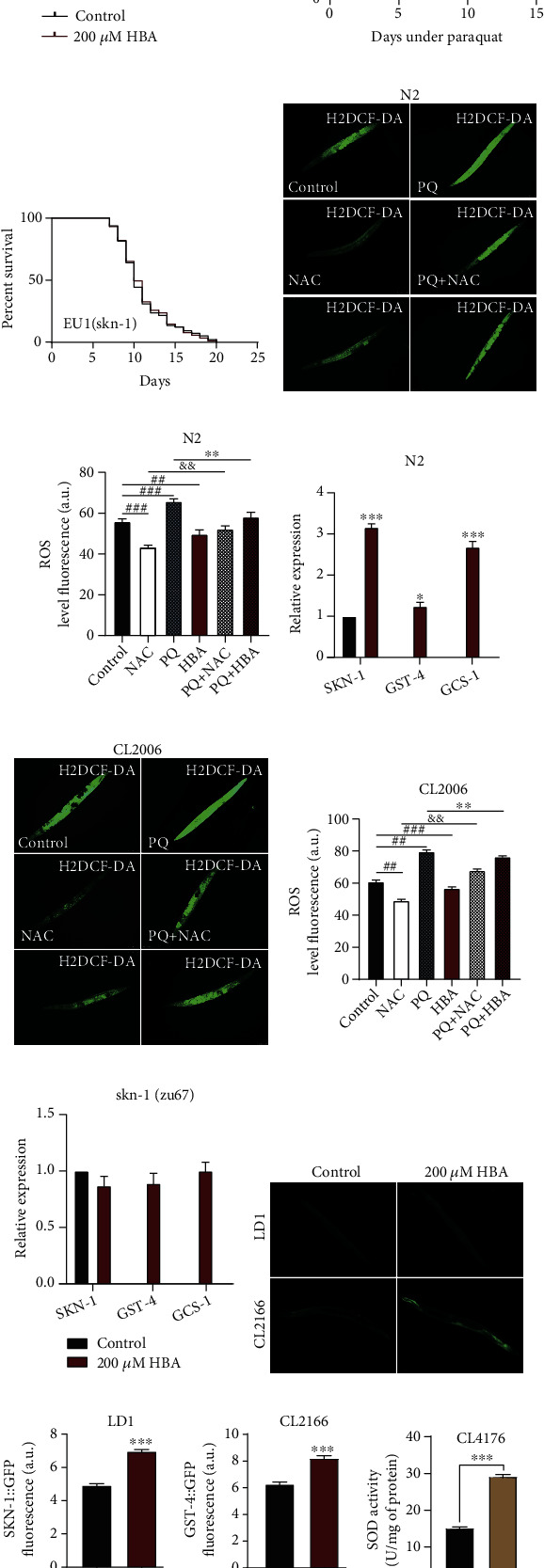
HBA enhances the antioxidant capacity of *C. elegans*. The lifespan expectancy of *C. elegans* wild-type (a) N2 and (b) strain CL2006 were fed with or without 200 *μ*M HBA for 7 days and then exposed to 20 mM of paraquat. (c) The lifespan expectancy of null mutant *skn-1(zu67)* fed with or without 200 *μ*M HBA (*p* > 0.05). The expression of *skn-1* and its target genes in wild-type (f) N2 and (i) mutant *skn-1(zu67)*. The CM-H_2_DCFDA staining picture of wild-type (d) N2 and (g) CL2006 was fed with or without HBA (200 *μ*M) or NAC (5 mM). The quantification of ROS levels indicated in pictures (e) and (h) using ImageJ (*p* < 0.001). (h) The green fluorescence pictures of *C. elegans* transgenic strain LD1 *(Is007) [Pskn-1::skn -1b/c::GFP; pRF4 rol-6 (su1006)])* (top) and CL2166 *(dvIs19)[(pAF15)gst-4p::GFP]* (bottom) fed with or without 200 *μ*M HBA. (j) The quantification of the fluorescence intensity of SKN-1::GFP (I) and GST-4:: GFP. (k) The superoxide dismutase (SOD) activity of strain CL4176 fed with 200 *μ*M HBA (*p* < 0.001). Prism 6.0 was used for statistical analysis, and the values were expressed as mean ± SEM. The *t*-test or log-rank test were used to calculate the statistical significance in *p* value (^∗^*p* < 0.05, ^∗∗^*p* < 0.01, and ^∗∗∗^*p* < 0.001). Statistical details and repeats of these assays are summarized in Table S6, S7, S8, and S9 (supplementary information).

Oxidative stress has long been associated with aging and age-related diseases [70]. Oxidative stress was reported to precede fibrillar deposition of Alzheimer's disease A*β* (1-42) in a transgenic *C. elegans* model [71]. The toxicity of A*β* could also induces unfolded protein stress. So, we investigate whether HBA could enhance the activities of cellular protective pathways to increase the resistance to various stresses, such as heat, oxidant, and pathogen. Paraquat is an oxidant inducing acute oxidative stress in cells. Our results showed that the HBA could increase the survival rate of wild-type N2 strain exposed to paraquat and prolong their lifespan by 20.39% (*p* < 0.001) (Figure 4(a)). Consistently, HBA also increased the survival rate of AD model of strain CL2006 exposed to paraquat and prolonged their lifespan by 21.05% (*p* < 0.001) (Figure 4(b)). We used H_2_DCF-DA as a free radical sensor to indicate the ROS content in worms. Our results showed that HBA treatment could significantly reduce the accumulation of ROS in wild-type N2 (Figures 4(d) and 4(e)) and AD model worms CL2006 (Figures 4(f) and 4(g)). It has been reported that the oxidation of A*β* protein itself has been found in amyloid plaques and SOD is the main enzyme that reduces the damage of superoxide anion in the body [72, 73]. So, we measured the SOD enzyme activity in strain CL4176. Our results showed that HBA significantly increased the SOD enzyme activity in strain CL4176 (Figure 4(k)). The SKN-1 transcription factor plays a critical role in oxidative stress response by regulating the expression of genes encoding phase II detoxification enzymes, such as glutathione S-transferases (GSTs) [74]. The *C. elegans* LD1 Is007 *(skn-1::GFP*) expresses fusion protein SKN-1::GFP (Figure 4(h)). Our results showed that HBA significantly increased the expression of SKN-1 in LD1 worms compared with the control worms (Figure 4(i)). In addition, the expression of glutathione S-transferase 4 (GST-4) also increased in the worms treated with HBA (Figures 4(h) and 4(j)). DAF-16/FOXO and SKN-1/Nrf2 are key transcription factors in the modulation of oxidative stress resistance and longevity in *C. elegans*. Our results showed that HBA could not extend the lifespan of the mutant *skn-1*(*zu67*) (Figure 4(c)). So, we detected the expression of downstream target genes of SKN-1 in N2 and the mutant *skn-1*(zu67). Our results showed that HBA treatment significantly increased the expression of SKN-1 and the downstream target genes in wild-type N2, but not in *skn-1* mutants (Figures 4(f) and 4(i)).

**Figure 5:**
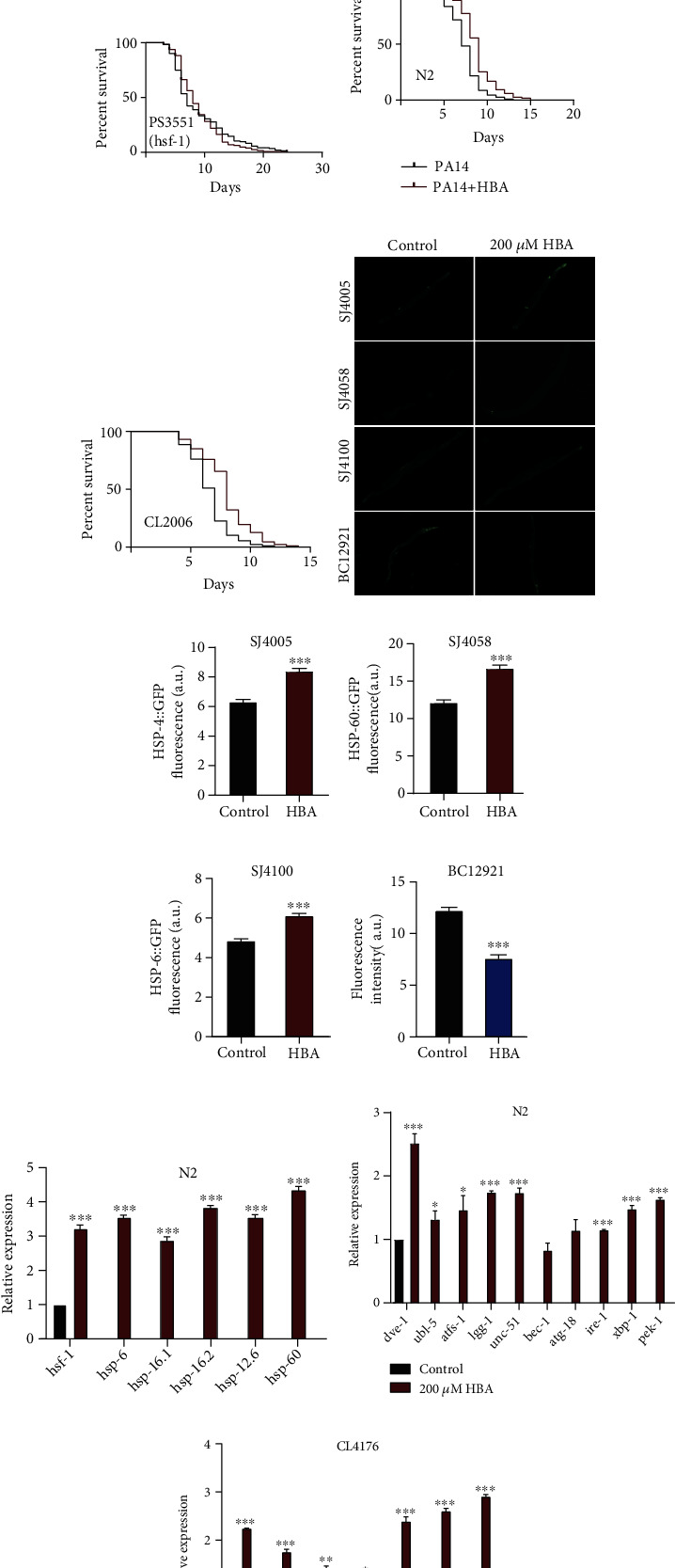
HBA enhances the stress resistance of *C. elegans*. The survival span of (a) wild-type N2 and (b) transgenic CL2006 under ambient temperature of 35°C after being treated with or without 200 *μ*M HBA for 7 days. (c) The lifespan expectancy of null mutant *hsf-1(sy441)* fed with or without 200 *μ*M HBA (*p* > 0.05). (f) The fluorescent pictures and the fluorescence quantification of *C. elegans* strain (from top to bottom) (g) SJ4005 *zcIs4 [hsp-4::GFP] V*, (h) SJ4058 *zcIs9 [hsp-60::GFP + lin-15(+)]*, (i) SJ4100 *zcIs13 [hsp-6p::GFP + lin-15(+)]*, (j) BC12921 *sIs10729 [rCes T12G3.1::GFP + pCeh361]* fed with or without 200 *μ*M HBA. The survival span of the wild-type (d) N2 and (e) CL2006 fed with pathogenic bacteria *Pseudomonas aeruginosa* (PA14) after being treated with or without 200 *μ*M HBA for 7 days. The results showed that HBA could significantly prolong the lifespan of N2 and CL2006 by 15.21% (*p* < 0.001) and 20.37% (*p* < 0.001), respectively. (k) The mRNA levels of genes in wild-type N2 fed with 200 *μ*M HBA (mean ± SD, *n* = 3). The mRNA levels of genes in wild-type (l) N2 and (m) CL4176 fed with 200 *μ*M HBA (mean ± SD, *n* = 3). Prism 6.0 was used for statistical analysis, and the values were expressed as mean ± SEM. Use *t*-test or log-rank test to express statistical significance in *p* value (^∗^*p* < 0.01, ^∗∗^*p* < 0.05, and ^∗∗∗^*p* < 0.001). Statistical details and repeats of these assays are summarized in Table S6, S9, S10, and S11 (supplementary information).

Heat shock could disrupt proteostasis, leading to protein misfolding and aggregation. The highly conserved transcription factor heat shock factor 1 (HSF-1) is a central regulator of heat shock response. In response to elevated temperatures, HSF-1 drives the transcription of genes, including chaperone heat-shock proteins (HSPs) to prevent heat stress-induced misfolding and aggregation of proteins. Our results showed that HBA enhanced the lifespan of wild-type N2 by 18.47% under heat stress (*p* < 0.001) (Figure 5(a)). HBA treatment prolonged the lifespan of strain CL2006 expressing A*β* constitutively by 26.46% under heat stress (*p* < 0.001) (Figure 5(b)). HBA significantly increased the mRNA levels of *hsf-1* and its targeted genes *hsp-6*, *hsp-16.1*, *hsp-16.2*, and *hsp-60* (Figure 5(k)). Furthermore, we tested the expression of heat shock proteins HSP-4 and HSP-60 conjugated with green fluorescent protein by determining the fluorescent intensity. HBA significantly increased the expression of proteins HSP-4 and HSP-60 (Figures 5(f)–5(h)). HSF-1 is an essential regulator of proteostasis, immunity, and aging [75, 76] and also acts downstream of the IIS signaling pathway [76]. We found that HBA could not extend the lifespan of *hsf-1* null mutant (Figure 5(c)).

The human bacterial pathogen *P*. *aeruginosa* (PA) secretes phenazine toxins disrupting mitochondrial function and exotoxin A interrupting translation [77]. The toxins of PA14 trigger the immune response, such as unfolded mitochondria protein response, endoplasmic stress response, and xenobiotic detoxification response. To analyze the antipathogenic effects of HBA, we explore the lifespan of wild-type N2 and strain CL2006 fed with PA14. Our results showed that HBA could improve the survival of wild-type N2 and AD strain CL2006 and extend the lifespan of N2 and CL2006 by 15.21% (*p* < 0.001) (Figure 5(d)) and 20.37% (*p* < 0.001), respectively (Figure 5(e)). The expression of chaperone protein HSP-6 is an indicator of mitochondrion stress response. So, we measured the fluorescent intensity of the transgenic strain SJ4100 expressing HSP-6 conjugated with GFP fed with HBA (Figure 5(f)). HBA treatment significantly increased protein expression of HSP-6 in SJ4100 (Figure 5(i)). HBA also significantly increased the mRNA level of *hsp-6* in wild-type N2 (Figure 5(k)). Furthermore, HBA significantly increased the mRNA levels of genes regulating endoplasmic unfolded protein response, such as ire-1, xbp-1, and perk-1, as well as mitochondrial unfolded protein response, such as *dve-1*, *ubl-5*, and *atfs-1* (Figures 5(l) and 5(m)).

Autophagy is a fundamental intracellular catabolic process critical for degradation, turnover, and renewal of excess or dysfunctional cellular components, aberrant protein depositions, and intracellular microorganisms via “xenophagy” [78]. The abnormality of autophagy is closely related to the development of age-related diseases such as Alzheimer's disease [79]. We found that HBA significantly increased the mRNA levels of autophagy-related genes such as *lgg-1 unc-51*, *bec-1*, and *atg-18* in CL4176 strain and wild-type N2 (Figures 5(l) and 5(m)). The *C. elegans* BC12921 expresses autophagy substrate protein SQST-1 conjugated with GFP driving by the sqst-1 promoter. As the autophagy activity increases, the degradation of SQST-1::GFP increases, so the green fluorescent intensity in worm BC12921 decreases [80]. Our results showed that HBA treatment significantly decreased the fluorescence intensity of BC12921 (Figures 5(f) and 5(j)).

### 3.4. The Effect of HBA on Nutrition Sensing and Metabolism

**Figure 6:**
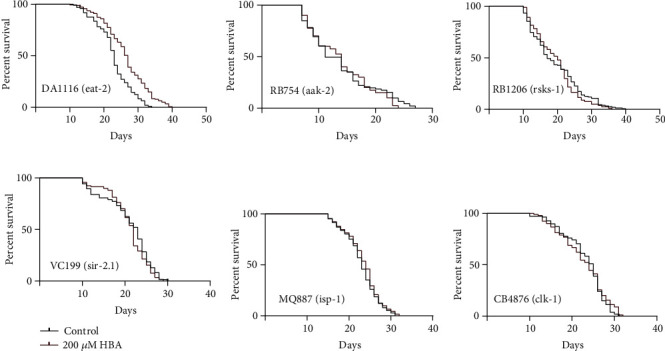
HBA requires FOXO/DAF-16 to extend the healthy lifespan of *C. elegans*. The lifespan analysis of *eat-2* null mutant *eat-2(ad1116) II* (a) (*p* < 0.05), null mutant *aak-2(ok524)* (b) (*p* > 0.05), *rsks-1(ok1255)* (c) (*p* > 0.05), *sir-2.1(ok434)* (d) (*p* > 0.05), *isp-1(qm150)* (e) (*p* > 0.05), and *clk-1(e2519)* (f) (*p* > 0.05) fed with 200 *μ*M HBA. Prism 6.0 was used for statistical analysis, and the values were expressed as mean ± SEM. Use *t*-test or log-rank test to express statistical significance in *p* value (^∗^*p* < 0.05). Statistical details and repeats of these assays are summarized in Table S10 (supplementary information).

The *eat-2(ad1116)* mutant has insufficient food uptake for the defect in pharyngeal function. We found that HBA can extend the lifespan of the *eat-2* mutant (Figure 6(a)). AMP-activated protein kinase (AMPK) is a critical energy censor regulating the activity of the target of rapamycin (TOR) [81]. The mutation (*aak-2(ok524)*) in *aak-2* (encoding a subunit of AMPK) inhibited the utilization of glucose and led to an extended lifespan [82]. Our results showed that HBA could not further prolong the lifespan of this mutant (Figure 6(b)). RSKS-1 is a homolog of S6 kinase (S6K), the downstream target of TOR. Our results showed that HBA cannot further extend the lifespan of the loss-of-function mutant *rsks-1(ok1255)* (Figure 6(c)). In addition, the current research results suggest that SIR-2.1 is an enzyme closely related to DR in *C. elegans* [83]. Our results showed that HBA also could not prolong the lifespan of the mutant *sir-2.1 (ok434)* (Figure 6(d)).

The gene *isp-1* encoding Rieske iron-sulfur polypeptide is involved in mitochondrial electron transport and can also activate the AMPK pathway by reducing the level of ATP. Therefore, we explored whether mitochondrial function plays an important role in the effects of HBA on lifespan extension and resistance to AD. ISP-1 mutant *isp-1(qm150)* and CLK-1 mutant *clk-1(e2519)* are both mutants of mitochondrial respiratory dysfunction. Our results showed that HBA could not extend the lifespan of these two mutants (Figures 6(e) and 6(f)).

## 4. Discussion

In this study, we found that *para*-hydroxybenzyl alcohol (HBA) could delay the progression of aging-related diseases, such as AD, PD, and HD. HBA could enhance the ability of *C. elegans* to resist pathogenic bacteria, oxidant, and heat shock stresses. HBA activates various cellular protective pathways, such as increased antioxidative and detoxification activities regulated by SKN-1 and DAF-16, increased protein homeostasis regulated by HSF-1, and increased mitochondrion regeneration and autophagy activities. The abovementioned effects of HBA may improve the physiological functions against aging and extend the lifespan of *C. elegans*.

The ability to resist stress is closely related to the ability to resist aging. We found that HBA could increase the expression of antioxidative and detoxification genes, such as *skn-1* downstream genes *gst-4* and *gcs-1* and *daf-16* downstream genes *sod-3*, *dod-3*, and *ctl-1*. HBA could not extend the lifespan of the null mutant of *skn-1*. Heat shock factor-1 (HSF-1) is an essential regulator of proteostasis, immunity, and aging [33, 34]. HBA increased the expression of *hsf-1* and its downstream factors like *hsp-6*, *hsp-16.1*, *hsp-16.2*, and *hsp-60*. We found that HBA also could not extend the lifespan of *hsf-1* null mutant. HBA could increase the expression of genes regulating the quality and mitochondria unfolded protein response. The loss-of-function mutants in genes encoding the mitochondrial respiration chain complex, such as *clk-1* and *isp-1*, live longer with activated mitochondria unfolded protein response. HBA could not further extend the long-live mutants of *clk-1* and *isp-1*. Furthermore, HBA could increase the expression of genes encoding components of autophagy, such as *bec-1*, *unc-51*, *lgg-1*, and *atg-18*. Autophagy is a fundamental process for cellular quality control by intracellular degradation cellular components and molecules and xenobiotics. The abovementioned results suggest that HBA extends the lifespan of *C. elegans* by activating multiple cellular protective pathways. Our study supports the previous report that HBA could prevent A*β*_1-42_ oligomer-induced synaptic and cognitive damage through Nrf2 in mice [46].

Nutritional sensing pathways play a very important role in aging and longevity [81]. Here, we also explored the relationship between HBA and energy-related pathways. HBA could not significantly extend the lifespan of long-lived mutants derived from nutrition-sensing pathway genes, such as *rsks-1* and *sir-2.1*, indicating either that the effect of HBA on prolonging lifespan is not enough to distinguish it from mutants that have extended longevity or that these genes are necessary for HBA to extend the lifespan of *C. elegans*. We also found that HBA could not extend the life of long-lived null mutants of the upstream gene of *daf-16*, such as the null mutants of *daf-2*, *akt-1*, and *akt-2*, indicating either that the effect of HBA on prolonging the lifespan is not enough to distinguish it from mutants that have extended-longevity or that the IIS pathway is a necessary for HBA to extend the life of *C. elegans*.

In conclusion, our study demonstrated that HBA activated multiple cellular protective pathways, such as SKN-1-regulated antioxidative and detoxification, HSF-1-regulated proteostasis maintenance, mitochondrion and endoplasmic stress response, and autophagy pathways (Figure S1). These improved activities protect *C. elegans* against various environmental stresses, aging, and aging-related diseases, extend the lifespan of *C. elegans*, and delayed the progression of neurodegenerative diseases. Our study suggests that HBA is a potential candidate for developing antiaging medicine and worth for further research on its pharmaceutical mechanism and applications.

## Data Availability

All the figures and tables used to support the findings of this study are included within the article and supplementary materials.

## Supplementary Materials

Supplementary Materials Figure S1: graphic abstract. Table S1: effect of *para*-hydroxybenzyl alcohol (HBA) on the paralysis of Alzheimer's disease (AD) *C. elegans* (CL4176). Table S2: HBA decreases the aggregation of neurodegenerative disease-associated proteins. Table S3: effect of HBA on the movement of wild-type *C. elegans* (N2) and AD *C. elegans* (CL2006). Table S4: effect of HBA on lipofuscin in wild-type *C. elegans* (N2) and AD *C. elegans* (CL2006). Table S5: effect of HBA on the chemotaxis of *C. elegans*. Table S6: effect of HBA on the stress resistance of wild-type *C. elegans* (N2) and AD *C. elegans* (CL2006). Table S7: effect of HBA on superoxide dismutase in AD *C. elegans* (CL4176). Table S8: effect of HBA on ROS accumulation in wild-type *C. elegans* (N2) and AD *C. elegans* (CL2006). Table S9: effect of HBA on the gene expression at the protein level showed as fluorescence intensity in *C. elegans*. Table S10: effect of HBA on the lifespan of *C. elegans*. Table S11: effect of HBA on gene expression at the mRNA levels in *C. elegans*. Table S12: primers used for the analysis of gene expression in *C. elegans*.
