# Supplementary material for: Para-Hydroxybenzyl Alcohol Delays the Progression of Neurodegenerative Diseases in Models of Caenorhabditis elegans through Activating Multiple Cellular Protective Pathways
Source: Oxid Med Cell Longev. 2022 Mar 31;2022:8986287. doi: 10.1155/2022/8986287 (PMC8989581; doi:10.1155/2022/8986287)
Supplement: Supplementary Materials — Figure S1: graphic abstract. Table S1: effect of para-hydroxybenzyl alcohol (HBA) on the paralysis of Alzheimer's disease (AD) C. elegans (CL4176). Table S2: HBA decreases the aggregation of neurodegenerative disease-associated proteins. Table S3: effect of HBA on the movement of wild-type C. elegans (N2) and AD C. elegans (CL2006). Table S4: effect of HBA on lipofuscin in wild-type C. elegans (N2) and AD C. elegans (CL2006). Table S5: effect of HBA on the chemotaxis of C. elegans. Table S6: effect of HBA on the stress resistance of wild-type C. elegans (N2) and AD C. elegans (CL2006). Table S7: effect of HBA on superoxide dismutase in AD C. elegans (CL4176). Table S8: effect of HBA on ROS accumulation in wild-type C. elegans (N2) and AD C. elegans (CL2006). Table S9: effect of HBA on the gene expression at the protein level showed as fluorescence intensity in C. elegans. Table S10: effect of HBA on the lifespan of C. elegans. Table S11: effect of HBA on gene expression at the mRNA levels in C. elegans. Table S12: primers used for the analysis of gene expression in C. elegans. [file 8986287.f1.docx]

***para*-Hydroxybenzyl Alcohol Delays the Progression of Neurodegenerative Diseases in Models of *Caenorhabditis elegans* through Activating Multiple Cellular Protective Pathways**

Yu Liu^1,2^, Yu-Yang Lu^1^, Lv Huang^1^, Lin Shi^1^, Zhuo-Ya Zheng^1^, Jian-Ning Chen^1^, Yuan Qu^1^, Hai-Ting Xiao^1^, Huai-Rong Luo^1,3,4*^, Gui-Sheng Wu^1,3,4,*^

^1^ Key Laboratory for Aging and Regenerative Medicine, Department of Pharmacology, School of Pharmacy, Southwest Medical University, Luzhou, Sichuan 646000, China

^2^ Department of Pharmacy, Daping Hospital, Army Medical University, Chongqing 400042, China

^3^ Central Nervous System Drug Key Laboratory of Sichuan Province, Luzhou, Sichuan 646000, China

^4^ Key Laboratory of Medical Electrophysiology, Ministry of Education & Medical Electrophysiological Key Laboratory of Sichuan, Institute of Cardiovascular Research, Southwest Medical University, Luzhou, Sichuan 646000, China

* Corresponding author: Dr. Huai-Rong Luo, Gui-sheng Wu

Key Laboratory for Aging and Regenerative Medicine, Department of Pharmacology, School of Pharmacy, Southwest Medical University, Luzhou, Sichuan 646000, China

Phone: +86 830-3160842; Fax: +86 830-3160842

E-mail address: [wgs@swmu.edu.cn](mailto:wgs@swmu.edu.cn), [lhr@swmu.edu.cn](mailto:lhr@swmu.edu.cn)

ORCID: <https://orcid.org/0000-0003-1913-9566>, <https://orcid.org/0000-0001-8912-6694>

**Supplementary materials**

**Contents:**

**Figure S1. Graphic abstract.**

**Table S1. Effect of p-hydroxybenzyl alcohol on the paralysis of AD *C. elegans* (CL4176).**

**Table S2. Effect of p-hydroxybenzyl alcohol on the aggregation of neurodegenerative diseases-associated proteins.**

**Table S3. Effect of p-hydroxybenzyl alcohol on movement of** **wild-type *C. elegans* (N2) and AD *C. elegans* (CL2006).**

**Table S4. Effect of p-hydroxybenzyl alcohol on lipofuscin in wild-type *C. elegans* (N2) and AD *C. elegans* (CL2006)*.***

**Table S5. Effect of p-hydroxybenzyl alcohol on the chemotaxis of *C. elegans*.**

**Table S6. Effect of p-hydroxybenzyl alcohol on the stress resistance of wild-type *C. elegans* (N2) and AD *C. elegans* (CL2006).**

**Table S7. Effect of p-hydroxybenzyl alcohol on superoxide dismutase in AD *C. elegans* (CL4176).**

**Table S8. Effect of p-hydroxybenzyl alcohol on ROS accumulation in wild-type *C. elegans* (N2) and AD *C. elegans* (CL2006).**

**Table S9. Effect of p-hydroxybenzyl alcohol on the fluorescence intensity of *C. elegans*.**

**Table S10. Effect of p-hydroxybenzyl alcohol on the lifespan of *C. elegans*.**

**Table S11. Effect of p-hydroxybenzyl alcohol on the mRNA levels of different genes in *C. elegans*.**

**Table S12. Primers used for the analysis of gene expression in *C. elegans*.**

**Figure S1. Graphic abstract.**


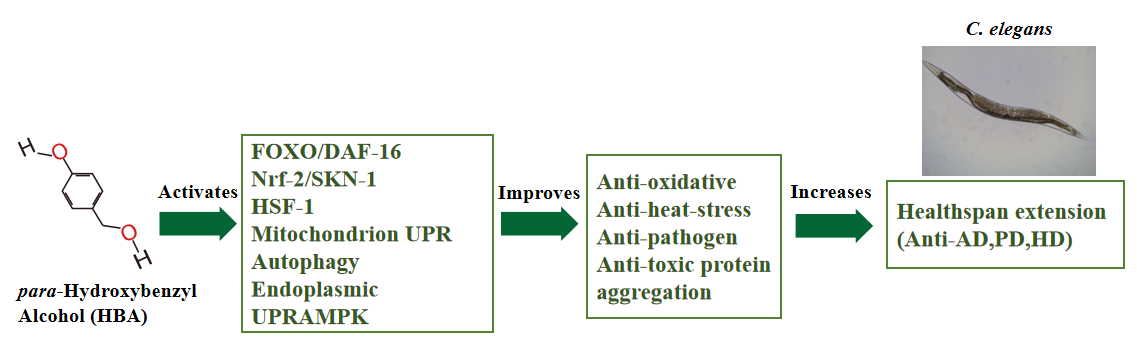


**Table S1. Effect of p-hydroxybenzyl alcohol on the paralysis of AD *C. elegans* (CL4176).**

| Figure | Strains | Treatments | Mean lifespan  ±SEM | *p* value  VS  Control | （%）  Change in  mean lifespan | N |
| --- | --- | --- | --- | --- | --- | --- |
| 1B | CL4176 25℃ | | | | | |
|  | EXP.1 | 25℃/Control | 35.3097±0.382 |  |  | 113 |
|  | EXP.1 | 25℃/50μM HBA | 36.6538±0.473 | *p*=0.113 | # | 104 |
|  | EXP.1 | 25℃/100μM HBA | 38.8602±0.567 | *p*<0.001 | 10.06 | 93 |
|  | EXP.1 | 25℃/200μM HBA | 42.6408±0.629 | *p*<0.001 | 20.76 | 103 |
|  | EXP.1 | 25℃/400μM HBA | 40.2553±0.666 | *p*<0.001 | 14.01 | 94 |
|  | EXP.2 | 25℃/Control | 34.7647±0.347 |  |  | 102 |
|  | EXP.2 | 25℃/50μM HBA | 36±0.428 | *p*=0.120 | # | 101 |
|  | EXP.2 | 25℃/100μM HBA | 37.9570±0.409 | *p*<0.001 | 9.18 | 93 |
|  | EXP.2 | 25℃/200μM HBA | 42.4255±0.584 | *p*<0.001 | 22.04 | 94 |
|  | EXP.2 | 25℃/400μM HBA | 39.7053±0.623 | P<0.001 | 14.21 | 95 |
|  | EXP.3 | 25℃/Control | 34.5370±0.348 |  |  | 108 |
|  | EXP.3 | 25℃/50μM HBA | 36.5825±0.461 | *p*=0.006 | 5.92 | 103 |
|  | EXP.3 | 25℃/100μM HBA | 38.7579±0.545 | *p*<0.001 | 12.22 | 95 |
|  | EXP.3 | 25℃/200μM HBA | 41.5876±0.668 | *p*<0.001 | 20.41 | 97 |
|  | EXP.3 | 25℃/400μM HBA | 39.6566±0.619 | *p*<0.001 | 14.82 | 99 |
| 1C | CL2006 |  |  |  |  |  |
|  | EXP.1 | 25℃/Control | 15.5488±0.569 |  |  | 82 |
|  | EXP.1 | 25℃/50μM HBA | 16.7059±0.396 | *p*=0.011 | 7.44 | 102 |
|  | EXP.1 | 25℃/100μM HBA | 17.2436±0.496 | *p*=0.002 | 10.90 | 78 |
|  | EXP.1 | 25℃/200μM HBA | 18.5814±0.466 | *p*<0.001 | 19.50 | 86 |
|  | EXP.1 | 25℃/400μM HBA | 18.1170±0.406 | *p*<0.001 | 16.52 | 94 |
|  | EXP.2 | 25℃/Control | 15.6364±0.584 |  |  | 77 |
|  | EXP.2 | 25℃/50μM HBA | 16.8947±0.374 | *p*=0.004 | 8.05 | 114 |
|  | EXP.2 | 25℃/100μM HBA | 17.5699±0.432 | *p*<0.001 | 12.37 | 93 |
|  | EXP.2 | 25℃/200μM HBA | 18.94±0.464 | *p*<0.001 | 21.13 | 100 |
|  | EXP.2 | 25℃/400μM HBA | 18.2784±0.415 | *p*<0.001 | 16.90 | 97 |
|  | EXP.3 | 25℃/Control | 15.1392±0.551 |  |  | 79 |
|  | EXP.3 | 25℃/50μM HBA | 16.7030±0.406 | *p*=0.001 | 10.33 | 101 |
|  | EXP.3 | 25℃/100μM HBA | 17.2697±0.458 | *p*<0.001 | 14.07 | 89 |
|  | EXP.3 | 25℃/200μM HBA | 18.4318±0.489 | *p*<0.001 | 21.75 | 88 |
|  | EXP.3 | 25℃/400μM HBA | 17.6118±0.454 | *p*<0.001 | 16.33 | 85 |

*p*-value was analyzed by log-rank (Mantel-Cox) test. N: number of dead worms.

*P* < 0.05 indicated that the experiment was statistically significant, while *p* > 0.05 indicated that the experiment was not statistically significant.

**Table S2. Effect of p-hydroxybenzyl alcohol on the aggregation of neurodegenerative diseases-associated proteins.**

| Figure | Strains | Treatments | Mean  protein aggregation  ± SEM | *p* value  VS  Control | N |
| --- | --- | --- | --- | --- | --- |
| 1E | CL2006 |  |  |  |  |
|  | EXP.1 | 20℃/Control | 7.99±0.19 |  | 92 |
|  | EXP.1 | 20℃/200μM HBA | 8.16±0.19 |  | 100 |
|  | EXP.2 | 20℃/Control | 8.24±0.171 |  | 106 |
|  | EXP.2 | 20℃/200μM HBA | 10.12±0.18 | *p*<0.001 | 102 |
|  | EXP.3 | 20℃/Control | 9.99±0.18 | *p*<0.001 | 115 |
|  | EXP.3 | 20℃/200μM HBA | 9.84±0.195 | *p*<0.001 | 117 |
| 2B | NL5901 |  |  |  |  |
|  | EXP.1 | 20℃/Control | 7.99±0.19 |  | 92 |
|  | EXP.1 | 20℃/200μM HBA | 8.16±0.19 |  | 100 |
|  | EXP.2 | 20℃/Control | 8.24±0.171 |  | 106 |
|  | EXP.2 | 20℃/200μM HBA | 10.12±0.18 | *p*<0.001 | 102 |
|  | EXP.3 | 20℃/Control | 9.99±0.18 | *p*<0.001 | 115 |
|  | EXP.3 | 20℃/200μM HBA | 9.84±0.195 | *p*<0.001 | 117 |
| 2F | AM141 |  |  |  |  |
|  | EXP.1 | 20℃/Control | 7.99±0.19 |  | 92 |
|  | EXP.1 | 20℃/200μM HBA | 8.16±0.19 |  | 100 |
|  | EXP.2 | 20℃/Control | 8.24±0.171 |  | 106 |
|  | EXP.2 | 20℃/200μM HBA | 10.12±0.18 | *p*<0.001 | 102 |
|  | EXP.3 | 20℃/Control | 9.99±0.18 | *p*<0.001 | 115 |
|  | EXP.3 | 20℃/200μM HBA | 9.84±0.195 | *p*<0.001 | 117 |
| 2D | BZ555 |  |  |  |  |
|  | EXP.1 | Control | 48.4188±0.8232 | *p*<0.001 | 30 |
|  | EXP.1 | Control/6-OHDA | 20.1681±0.6542 | *p*<0.001 | 30 |
|  | EXP.1 | Control/6-OHDA/200μM HBA | 30.1516±1.0199 | # | 30 |
|  | EXP.1 | Control/6-OHDA/2mM Levodopa | 36.3922±0.5745 | *p*<0.001 | 30 |
|  | EXP.2 | Control | 48.0112±0.7781 | *p*<0.001 | 30 |
|  | EXP.2 | Control/6-OHDA | 20.1681±0.4982 | *p*<0.001 | 30 |
|  | EXP.2 | Control/6-OHDA/200μM HBA | 30.8697±0.7712 | # | 30 |
|  | EXP.2 | Control/6-OHDA/2mM Levodopa | 35.1882±0.6542 | *p*<0.001 | 30 |
|  | EXP.3 | Control | 48.4915±0.9131 | *p*<0.001 | 30 |
|  | EXP.3 | Control/6-OHDA | 20.1681±0.4982 | *p*<0.001 | 30 |
|  | EXP.3 | Control/6-OHDA/200μM HBA | 29.2613±0.8020 | # | 30 |
|  | EXP.3 | Control/6-OHDA/2mM Levodopa | 35.7848±0.5560 | *p*<0.001 | 30 |

**Table S3. Effect of p-hydroxybenzyl alcohol on movement of wild-type *C. elegans* (N2) and AD *C. elegans* (CL2006).**

| Figure |  | Strains | Treatments | Mean Body movements/20s±SEM | *p* value  VS  Control | N |
| --- | --- | --- | --- | --- | --- | --- |
| 3A |  | N2(3DAY) |  |  |  |  |
|  |  | EXP.1 | 20℃/Control | 16.67±0.26 |  | 30 |
|  |  | EXP.1 | 20℃/200μM HBA | 20.27±0.27 | *p*<0.001 | 30 |
|  |  | EXP.2 | 20℃/Control | 17.07±0.26 |  | 30 |
|  |  | EXP.2 | 20℃/200μM HBA | 20.27±0.27 | *p*<0.001 | 30 |
|  |  | EXP.3 | 20℃/Control | 17.05±0.24 |  | 30 |
|  |  | EXP.3 | 20℃/200μM HBA | 20.20±0.28 | *p*<0.001 | 30 |
|  |  | N2(7DAY) |  |  |  |  |
|  |  | EXP.1 | 20℃/Control | 7.57±0.34 |  | 30 |
|  |  | EXP.1 | 20℃/200μM HBA | 11.57±0.3 | *p*<0.001 | 30 |
|  |  | EXP.2 | 20℃/Control | 7.57±0.34 |  | 30 |
|  |  | EXP.2 | 20℃/200μM HBA | 11.73±0.25 | *p*<0.001 | 30 |
|  |  | EXP.3 | 20℃/Control | 6.37±0.35 |  | 30 |
|  |  | EXP.3 | 20℃/200μM HBA | 11.93±0.32 | *p*<0.001 | 30 |
| 3B |  | CL2006(3DAY) |  |  |  |  |
|  |  | EXP.1 | 20℃/Control | 14.7±0.31 |  | 30 |
|  |  | EXP.1 | 20℃/200μM HBA | 20.3±0.29 | *p*<0.001 | 30 |
|  |  | EXP.2 | 20℃/Control | 15.23±0.29 |  | 30 |
|  |  | EXP.2 | 20℃/200μM HBA | 19.2±0.29 | *p*<0.001 | 30 |
|  |  | EXP.3 | 20℃/Control | 14.2±0.31 |  | 30 |
|  |  | EXP.3 | 20℃/200μM HBA | 19.57±0.31 | *p*<0.001 | 30 |
|  |  | CL2006(7DAY) |  |  |  |  |
|  |  | EXP.1 | 20℃/Control | 7.27±0.28 |  | 30 |
|  |  | EXP.1 | 20℃/200μM HBA | 10.43±0.39 | *p*<0.001 | 30 |
|  |  | EXP.2 | 20℃/Control | 6.73±0.25 |  | 30 |
|  |  | EXP.2 | 20℃/200μM HBA | 9.33±0.42 | *p*<0.001 | 30 |
|  |  | EXP.3 | 20℃/Control | 7.7±0.40 |  | 30 |
|  |  | EXP.3 | 20℃/200μM HBA | 10.17±0.29 | *p*<0.001 | 30 |

The body movement count time was 20 seconds per *C. elegans*. N was the experimental sample size, and *p* value was calculated by two-tailed t-test. *p*<0.05 indicated that the experiment was statistically significant

**Table S4. Effect of p-hydroxybenzyl alcohol on lipofuscin in wild-type *C. elegans* (N2) and AD *C. elegans* (CL2006)*.***

| Figure | Strains | Treatments | | Mean  Lipofuscin ± SEM | | *p*  value  VS  Control | | N | | |
| --- | --- | --- | --- | --- | --- | --- | --- | --- | --- | --- |
| 3D | N2 | |  | |  | |  | |  |  |
|  | EXP.1 | | 20℃/Control | | 15.97±0.21 | |  | | 30 |  |
|  | EXP.1 | | 20℃/200μM HBA | | 10.49±0.26 | | *p*<0.001 | | 30 |  |
|  | EXP.2 | | 20℃/Control | | 15.80±0.19 | |  | | 30 |  |
|  | EXP.2 | | 20℃/200μM HBA | | 10.22±0.54 | | *p*<0.001 | | 30 |  |
|  | EXP.3 | | 20℃/Control | | 16.26±0.18 | |  | | 30 |  |
|  | EXP.3 | | 20℃/200μM HBA | | 10.49±0.24 | | *p*<0.001 | | 30 |  |
| 3E | CL2006 | |  | |  | |  | |  |  |
|  | EXP.1 | | 20℃/Control | | 14.21±0.37 | |  | | 30 |  |
|  | EXP.1 | | 20℃/200μM HBA | | 6.07±0.35 | | *p*<0.001 | | 30 |  |
|  | EXP.2 | | 20℃/Control | | 14.68±0.36 | |  | | 30 |  |
|  | EXP.2 | | 20℃/200μM HBA | | 6.12±0.33 | | *p*<0.001 | | 30 |  |
|  | EXP.3 | | 20℃/Control | | 14.34±0.35 | |  | | 30 |  |
|  | EXP.3 | | 20℃/200μM HBA | | 5.71±0.29 | | *p*<0.001 | | 30 |  |

*p* value was determined by comparison between the control group and the experimental group in each independent experiment. The experimental data were processed and analyzed by SPSS 26.0 with Kaplan-Meier survival curve. *p* value was obtained by log-rank (Mantel-Cox) test analysis.

**Table S5. Effect of p-hydroxybenzyl alcohol on the chemotaxis of *C. elegans*.**

| Figure | Strains | | Treatments | | Mean  Chemotactic Index ± SEM | | *p* value  VS  Control | | | N | |
| --- | --- | --- | --- | --- | --- | --- | --- | --- | --- | --- | --- |
| 1G | | Cl2122 | |  | |  | |  |  | |  |
|  | | EXP.1 | | Control | |  | |  | 86 | |  |
|  | | EXP.1 | | Control | | 0.5021±0.0095 | |  | 89 | |  |
|  | | EXP.1 | | Control | |  | |  | 86 | |  |
|  | | EXP.1 | | 200μM HBA | |  | |  | 95 | |  |
|  | | EXP.1 | | 200μM HBA | | 0.5192±0.0236 | |  | 85 | |  |
|  | | EXP.1 | | 200μM HBA | |  | | *#* | 81 | |  |
|  | | EXP.2 | | Control | |  | |  | 81 | |  |
|  | | EXP.2 | | Control | | 0.4743±0.0048 | |  | 86 | |  |
|  | | EXP.2 | | Control | |  | |  | 84 | |  |
|  | | EXP.2 | | 200μM HBA | |  | |  | 91 | |  |
|  | | EXP.2 | | 200μM HBA | | 0.5060±0.0278 | |  | 82 | |  |
|  | | EXP.2 | | 200μM HBA | |  | | *#* | 81 | |  |
|  | | EXP.3 | | Control | |  | |  | 84 | |  |
|  | | EXP.3 | | Control | | 0.5192±0.0117 | |  | 87 | |  |
|  | | EXP.3 | | Control | |  | |  | 87 | |  |
|  | | EXP.3 | | 200μM HBA | |  | |  | 93 | |  |
|  | | EXP.3 | | 200μM HBA | | 0.5138±0.0493 | |  | 82 | |  |
|  | | EXP.3 | | 200μM HBA | |  | | # | 82 | |  |
|  | | CL2355 | |  | |  | |  |  | |  |
|  | | EXP.1 | | Control | |  | |  | 55 | |  |
|  | | EXP.1 | | Control | | 0.1744±0.0093 | |  | 57 | |  |
|  | | EXP.1 | | Control | |  | |  | 60 | |  |
|  | | EXP.1 | | 200μM HBA | |  | |  | 89 | |  |
|  | | EXP.1 | | 200μM HBA | | 0.3247±0.0104 | |  | 98 | |  |
|  | | EXP.1 | | 200μM HBA | |  | | *p*<0.001 | 76 | |  |
|  | | EXP.2 | | Control | |  | |  | 55 | |  |
|  | | EXP.2 | | Control | | 0.1567±0.0344 | |  | 58 | |  |
|  | | EXP.2 | | Control | |  | | *p*=0.009 | 54 | |  |
|  | | EXP.2 | | 200μM HBA | |  | |  | 88 | |  |
|  | | EXP.2 | | 200μM HBA | |  | |  | 98 | |  |
|  | | EXP.2 | | 200μM HBA | | 0.3303±0.0154 | | *p*=0.001 | 78 | |  |
|  | | EXP.3 | | Control | |  | |  | 53 | |  |
|  | | EXP.3 | | Control | |  | |  | 56 | |  |
|  | | EXP.3 | | Control | | 10.1597±0.0085 | |  | 60 | |  |
|  | | EXP.3 | | 200μM HBA | |  | |  | 88 | |  |
|  | | EXP.3 | | 200μM HBA | |  | |  | 82 | |  |
|  | | EXP.3 | | 200μM HBA | | 0.3305±0.0215 | | *p*=0.002 | 73 | |  |

**Table S6. Effect of p-hydroxybenzyl alcohol on the stress resistance of wild-type *C. elegans* (N2) and AD *C. elegans* (CL2006).**

| Figure | Strains | Treatments | Mean  Lifespan ± SEM | *p* value  VS  Control | （%）  Change in  mean lifespan | N |
| --- | --- | --- | --- | --- | --- | --- |
| 4A | N2 20℃ | | | | | |
|  | EXP.1 | Control/20Mm Paraquat | 8.05±0.269 |  |  | 93 |
|  | EXP.1 | 200μM HBA/20Mm Paraquat | 9.76±0.283 | *p*<0.001 | 21.15% | 103 |
|  | EXP.2 | Control/20Mm Paraquat | 8.06±0.272 |  |  | 98 |
|  | EXP.2 | 200μM HBA/20Mm Paraquat | 9.76±0.294 | *p*<0.001 | 21.09% | 113 |
|  | EXP.3 | Control/20Mm Paraquat | 7.64±0.281 |  |  | 108 |
|  | EXP.3 | 200μM HBA/20Mm Paraquat | 9.22±0.293 | *p*<0.001 | 20.75% | 116 |
| 4B | CL2006 | 20℃ |  |  |  |  |
|  | EXP.1 | Control/20Mm Paraquat | 6.60±0.257 |  |  | 80 |
|  | EXP.1 | 200μM HBA/20Mm Paraquat | 7.94±0.273 | *p*<0.001 | 20.23% | 93 |
|  | EXP.2 | Control/20Mm Paraquat | 6.71±0.237 |  |  | 75 |
|  | EXP.2 | 200μM HBA/20Mm Paraquat | 8.23±0.293 | *p*<0.001 | 22.68% | 79 |
|  | EXP.3 | Control/20Mm Paraquat | 6.15±0.272 |  |  | 80 |
|  | EXP.3 | 200μM HBA/20Mm Paraquat | 7.44±0.292 | *p*<0.001 | 20.90% | 85 |
| 5D | N2 | 20℃ |  |  |  |  |
|  | EXP.1 | Control/PA14 | 7.34±0.167 |  |  | 116 |
|  | EXP.1 | 200μM HBA/PA14 | 8.46±0.195 | *p*<0.001 | 15.21% | 119 |
|  | EXP.2 | Control/PA14 | 7.47±0.153 |  |  | 97 |
|  | EXP.2 | 200μM HBA/PA14 | 8.74±0.198 | *p*<0.001 | 16.93% | 119 |
|  | EXP.3 | Control/PA14 | 7.33±0.166 |  |  | 109 |
|  | EXP.3 | 200μM HBA/PA14 | 8.46±0.2 | *p*<0.001 | 15.36% | 103 |
| 5E | CL2006 |  |  |  |  |  |
|  | EXP.1 | Control/PA14 | 6.58±0.175 |  |  | 88 |
|  | EXP.1 | 200μM HBA/PA14 | 7.92±0.228 | *p*<0.001 | 20.37% | 87 |
|  | EXP.2 | Control/PA14 | 6.69±0.174 |  |  | 93 |
|  | EXP.2 | 200μM HBA/PA14 | 8.09±0.219 | *p*<0.001 | 20.90% | 93 |
|  | EXP.3 | Control/PA14 | 6.85±0.174 |  |  | 103 |
|  | EXP.3 | 200μM HBA/PA14 | 8.25±0.23 | *p*<0.001 | 20.33% | 109 |
| 5A | N2 |  |  |  |  |  |
|  | EXP.1 | Control/35℃ | 10.81±0.307 |  |  | 109 |
|  | EXP.1 | 200μM HBA/35℃ | 12.80±0.363 | *p*<0.001 | 18.48% | 92 |
|  | EXP.2 | Control/35℃ | 10.95±0.297 |  |  | 109 |
|  | EXP.2 | 200μM HBA/35℃ | 12.87±0.346 | *p*<0.001 | 17.48% | 99 |
|  | EXP.3 | Control/35℃ | 10.39±0.324 |  |  | 113 |
|  | EXP.3 | 200μM HBA/35℃ | 7.78±0.196 | *p*<0.001 | 18.17% | 101 |
| 5B | CL2006 | 20℃ |  |  |  |  |
|  | EXP.1 | Control/35℃ | 9.81±0.29 |  |  | 108 |
|  | EXP.1 | 200μM HBA/35℃ | 12.41±0.29 | *p*<0.001 | 26.46% | 102 |
|  | EXP.2 | Control/35℃ | 9.91±0.28 |  |  | 111 |
|  | EXP.2 | 200μM HBA/35℃ | 12.21±0.303 | *p*<0.001 | 24.36% | 107 |
|  | EXP.3 | Control/35℃ | 9.88±0.285 |  |  | 113 |
|  | EXP.3 | 200μM HBA/35℃ | 12.21±0.314 | *p*<0.001 | 24.42% | 104 |

*p* value was determined by comparison between the control group and the experimental group in each independent experiment. The data in the experimental group were counted as the number of normal dead nematodes. The experimental data were processed and analyzed by SPSS26.0 with Kaplan-Meier survival curve. *p* value was obtained by log-rank test analysis.

N: number of dead nematodes.

**Table S7. Effect of p-hydroxybenzyl alcohol on superoxide dismutase in AD *C. elegans* (CL4176).**

| Figure |  | | | Strains | Treatments | SOD activity  ± SEM | P value  VS  Control | N |
| --- | --- | --- | --- | --- | --- | --- | --- | --- |
| 1N | |  | CL4176 | |  |  |  |  |
|  | |  | EXP.1 | | 20℃/Control | 15.181±0.3012 |  | 3 |
|  | |  | EXP.1 | | 20℃/200μM HBA | 29.189±0.5522 | *p*<0.001 | 3 |
|  | |  | EXP.2 | | 20℃/Control | 15.178±0.1137 |  | 3 |
|  | |  | EXP.2 | | 20℃/200μM HBA | 29.180±0.2090 | *p*<0.001 | 3 |
|  | |  | EXP.3 | | 20℃/Control | 15.182±0.3012 |  | 3 |
|  | |  | EXP.3 | | 20℃/200μM HBA | 30.471±0.4413 | *p*<0.001 | 3 |

The experimental data were processed and analyzed by SPSS26.0, represented by Kaplan-Meier survival curve. *p* values were analyzed by Log-rank test.

**Table S8. Effect of p-hydroxybenzyl alcohol on ROS accumulation in wild-type *C. elegans* (N2) and AD *C. elegans* (CL2006).**

| Figure | Strains | Treatments | Mean  ROS level intensity  ± SEM | *p* value  VS  Control | N |
| --- | --- | --- | --- | --- | --- |
| 4E | N2 |  |  |  |  |
|  | EXP.1 | 20℃/Control | 55.954±1.3379 |  | 30 |
|  | EXP.1 | 20℃/200μM HBA | 11.219±2.048 | *p*=0.014 | 30 |
|  | EXP.1 | 20℃/1mM NAC | 43.506±0.7775 |  | 30 |
|  | EXP.1 | 20℃/2mM PQ | 65.810±0.1.210 |  | 30 |
|  | EXP.1 | 20℃/2mM PQ +200μMHBA | 52.278±1.5780 |  | 30 |
|  | EXP.1 | 20℃/2mM PQ +1mM NAC | 58.151±2.2910 |  | 30 |
|  | EXP.2 | 20℃/Control | 61.898±0.8232 |  | 30 |
|  | EXP.2 | 20℃/200μM HBA | 56.0±0.1.7333 | *p*=0.004 | 30 |
|  | EXP.2 | 20℃/1mM NAC | 43.999±0.6083 |  | 30 |
|  | EXP.2 | 20℃/2mM PQ | 67.910±0.4768 |  | 30 |
|  | EXP.2 | 20℃/2mM PQ +200μMHBA | 52.062±1.5812 |  | 30 |
|  | EXP.2 | 20℃/2mM PQ +1mM NAC | 58.416±2.1589 |  | 30 |
|  | EXP.3 | 20℃/Control | 63.008±0.8785 |  | 30 |
|  | EXP.3 | 20℃/200μM HBA | 57.478±0.6083 | *p*=0.002 | 30 |
|  | EXP.3 | 20℃/1mM NAC | 43.999±0.1137 |  | 30 |
|  | EXP.3 | 20℃/2mM PQ | 67.369±0.515 |  | 30 |
|  | EXP.3 | 20℃/2mM PQ +200μMHBA | 58.703±2.0369 |  | 30 |
|  | EXP.3 | 20℃/2mM PQ +1mM NAC | 48.597±0.5645 |  | 30 |
| 4G | CL2006 |  |  |  |  |
|  | EXP.1 | 20℃/Control | 60.949±0.9618 |  | 30 |
|  | EXP.1 | 20℃/200μM HBA | 56.814±0.8523 | *p*=0.002 | 30 |
|  | EXP.1 | 20℃/1mM NAC | 49.174±0.7864 |  | 30 |
|  | EXP.1 | 20℃/2mM PQ | 79.702±0.9843 |  | 30 |
|  | EXP.1 | 20℃/2mM PQ +200μM HBA | 67.977±0.9122 |  | 30 |
|  | EXP.1 | 20℃/2mM PQ +1mM NAC | 76.427±0.6544 |  | 30 |
|  | EXP.2 | 20℃/Control | 60.223±1.1753 |  | 30 |
|  | EXP.2 | 20℃/200μM HBA | 56.315±0.9174 | *p*=0.011 | 30 |
|  | EXP.2 | 20℃/1mM NAC | 48.362±0.8883 |  | 30 |
|  | EXP.2 | 20℃/2mM PQ | 79.501±1.1296 |  | 30 |
|  | EXP.2 | 20℃/2mM PQ +200μMHBA | 67.596±0.9259 |  | 30 |
|  | EXP.2 | 20℃/2mM PQ +1mM NAC | 75.494±0.5490 |  | 30 |
|  | EXP.3 | 20℃/Control | 59.836±1.0696 |  | 30 |
|  | EXP.3 | 20℃/200μM HBA | 56.62±0.9706 | *p*=0.030 | 30 |
|  | EXP.3 | 20℃/1mM NAC | 48.362±0.8883 |  | 30 |
|  | EXP.3 | 20℃/2mM PQ | 79.945±1.2119 |  | 30 |
|  | EXP.3 | 20℃/2mM PQ +200μM HBA | 68.098±0.8799 |  | 30 |
|  | EXP.3 | 20℃/2mM PQ +1mM NAC | 74.732±0.4680 |  | 30 |

The experimental data were processed and analyzed by SPSS26.0, represented by Kaplan-Meier survival curve. *P* values were analyzed by Log-rank test.

**Table S9. Effect of p-hydroxybenzyl alcohol on the fluorescence intensity of *C. elegans*.**

| Figure | Strains | Treatments | Mean  Fluorescence intensity ± SEM | P value  VS  Control | N | |
| --- | --- | --- | --- | --- | --- | --- |
| 3N | CF1553 |  |  |  | |  |
|  | EXP.1 | Control/20℃ | 6.2662±0.1344 |  | 30 | |
|  | EXP.1 | 200μM HBA /20℃ | 8.2294±0.1283 | *p*<0.001 | 30 | |
|  | EXP.2 | Control/20℃ | 6.0051±0.1007 |  | 30 | |
|  | EXP.2 | 200μM HBA /20℃ | 7.9490±0.1104 | *p*<0.001 | 30 | |
|  | EXP.3 | Control/20℃ | 6.2180±0.0974 |  | 30 | |
|  | EXP.3 | 200μM HBA /20℃ | 8.0369±0.1132 | *p*<0.001 | 30 | |
| 4J | CL2166 |  |  |  |  | |
|  | EXP.1 | Control/20℃ | 6.2037±0.2262 |  | 30 | |
|  | EXP.1 | 200μM HBA /20℃ | 8.1901±0.2016 | *p*<0.001 | 30 | |
|  | EXP.2 | Control/20℃ | 5.8666±0.2268 |  | 30 | |
|  | EXP.2 | 200μM HBA /20℃ | 7.8879±0.2241 | *p*<0.001 | 30 | |
|  | EXP.3 | Control/20℃ | 6.9877±0.1125 |  | 30 | |
|  | EXP.3 | 200μM HBA /20℃ | 7.8879±0.2241 | *p*<0.001 | 30 | |
| 5H | SJ4058 |  |  |  |  | |
|  | EXP.1 | Control/20℃ | 12.1521±0.3599 |  | 30 | |
|  | EXP.1 | 200μM HBA /20℃ | 16.7405±0.4228 | *p*<0.001 | 30 | |
|  | EXP.2 | Control/20℃ | 11.9575±0.3700 |  | 30 | |
|  | EXP.2 | 200μM HBA /20℃ | 15.2396±0.4497 | *p*<0.001 | 30 | |
|  | EXP.3 | Control/20℃ | 11.8006±0.3902 |  | 30 | |
|  | EXP.3 | 200μM HBA /20℃ | 15.6351±0.5391 | *p*<0.001 | 30 | |
| 5I | SJ4100 |  |  |  |  | |
|  | EXP.1 | Control/20℃ | 4.8584±0.0928 |  | 30 | |
|  | EXP.1 | 200μM HBA /20℃ | 6.1246±0.1088 | *p*<0.001 | 30 | |
|  | EXP.2 | Control/20℃ | 5.1134±0.1213 |  | 30 | |
|  | EXP.2 | 200μM HBA /20℃ | 6.0982±0.1194 | *p*<0.001 | 30 | |
|  | EXP.3 | Control/20℃ | 5.0062±0.1115 |  | 30 | |
|  | EXP.3 | 200μM HBA /20℃ | 5.9092±0.1198 | *p*<0.001 | 30 | |
| 5G | SJ4005 |  |  |  |  | |
|  | EXP.1 | Control/20℃ | 6.3182±0.1638 |  | 30 | |
|  | EXP.1 | 200μM HBA /20℃ | 8.4072±0.1799 | *p*<0.001 | 30 | |
|  | EXP.2 | Control/20℃ | 6.3182±0.1638 |  | 30 | |
|  | EXP.2 | 200μM HBA /20℃ | 8.5109±0.1527 | *p*<0.001 | 30 | |
|  | EXP.3 | Control/20℃ | 6.9765±0.1402 |  | 30 | |
|  | EXP.3 | 200μM HBA /20℃ | 8.5068±0.1447 | *p*<0.001 | 30 | |
| 4I | LD1 | 20℃ |  |  |  | |
|  | EXP.1 | Control/20℃ | 6.9673±0.1073 |  | 30 | |
|  | EXP.1 | 200μM HBA /20℃ | 4.912±0.1069 | *p*<0.001 | 30 | |
|  | EXP.2 | Control/20℃ | 6.9470±0.0933 |  | 30 | |
|  | EXP.2 | 200μM HBA /20℃ | 4.9428±0.1135 | *p*<0.001 | 30 | |
|  | EXP.3 | Control/20℃ | 6.9877±0.1125 |  | 30 | |
|  | EXP.3 | 200μM HBA /20℃ | 5.0118±0.1103 | *p*<0.001 | 30 | |
| 5J | BC12921 |  |  |  |  | |
|  | EXP.1 | Control/20℃ | 12.2484±0.2598 |  | 30 | |
|  | EXP.1 | 200μM HBA /20℃ | 7.6074±0.3460 | *p*<0.001 | 30 | |
|  | EXP.2 | Control/20℃ | 11.8438±0.3066 |  | 30 | |
|  | EXP.2 | 200μM HBA /20℃ | 7.4876±0.2751 | *p*<0.001 | 30 | |
|  | EXP.3 | Control/20℃ | 12.5478±0.3144 |  | 30 | |
|  | EXP.3 | 200μM HBA /20℃ | 7.9122±0.2679 | *p*<0.001 | 30 | |

**Table S10. Effect of p-hydroxybenzyl alcohol on the lifespan of *C. elegans*.**

| Figure | Strains | Treatments | Mean  Lifespan ± SEM | *p* value  VS  Control | | （%）  Change in  mean lifespan | N |
| --- | --- | --- | --- | --- | --- | --- | --- |
| 3G | N2 | | | | | | |
|  | EXP.1 | Control | 17.172±0.322 |  | |  | 116 |
|  | EXP.2 | Control | 16.9576±0.302 |  | |  | 118 |
|  | EXP.3 | Control | 16.5043±0.238 |  | |  | 117 |
|  | EXP.1 | 20℃/200μM HBA | 21.8081±0.375 | *p*<0.001 | | 26.99 | 99 |
|  | EXP.2 | 20℃/200μM HBA | 21.4835±0.379 | *p*<0.001 | | 26.69 | 91 |
|  | EXP.3 | 20℃/200μM HBA | 20.0879±0.38 | *p*<0.001 | | 21.71 | 91 |
| CF1038/*daf-16*(*mu86*) *Ⅰ* | | | | | | | |
| 3J | EXP.1 | Control | 16.7158±0.422 |  | |  | 95 |
|  | EXP.2 | Control | 16.7672±0.336 |  | |  | 116 |
|  | EXP.3 | Control | 16.8±0.406 |  | |  | 90 |
|  | EXP.1 | 20℃/200μM HBA | 17.5579±0.409 | # | | 5.038 | 95 |
|  | EXP.2 | 20℃/200μM HBA | 17.4946±0.338 | # | | 4.338 | 93 |
|  | EXP.3 | 20℃/200μM HBA | 17.8172±0.36 | # | | 6.055 | 93 |
| CB1370/*daf-2*(*e1370*) *Ⅲ* | | | | | | | |
| 3H | EXP.1 | Control | 45.0382±0.904 |  | |  | 157 |
|  | EXP.2 | Control | 44.4771±0.867 |  | |  | 153 |
|  | EXP.3 | Control | 43.7290±0.862 |  | |  | 155 |
|  | EXP.1 | 20℃/200μM HBA | 47.3333±0.951 | # | | -1.57 | 138 |
|  | EXP.2 | 20℃/200μM HBA | 44.808±0.969 | # | | 0.74 | 125 |
|  | EXP.3 | 20℃/200μM HBA | 43.68±0.99 | # | | -0.11 | 100 |
| RB759/*akt-1*(*ok525*) *Ⅴ* | | | | | | | |
| 3K | EXP.1 | Control | 21.9802±0.479 |  | |  | 101 |
|  | EXP.2 | Control | 22.6610±0.552 |  | |  | 118 |
|  | EXP.3 | Control | 21.3739±0.492 |  | |  | 115 |
|  | EXP.1 | 20℃/200μM HBA | 22.12±0.478 | # | | 0.64 | 100 |
|  | EXP.2 | 20℃/200μM HBA | 23.2908±0.463 | # | | 2.78 | 141 |
|  | EXP.3 | 20℃/200μM HBA | 20.6842±0.525 | # | | -3.23 | 133 |
| VC204/*akt-2*(*ok393*) *Ⅹ* | | | | | | | |
| 3L | EXP.1 | Control | 21.3494±0.583 |  | |  | 83 |
|  | EXP.2 | Control | 22.1154±0.628 |  | |  | 78 |
|  | EXP.3 | Control | 20.3387±0.432 |  | |  | 124 |
|  | EXP.1 | 20℃/200μM HBA | 20.875±0.515 | # | | -2.22 | 88 |
|  | EXP.2 | 20℃/200μM HBA | 22.2111±0.559 | # | | 0.43 | 90 |
|  | EXP.3 | 20℃/200μM HBA | 21.1053±0.411 | # | | 3.77 | 133 |
| PS3551*/hsf-1*(*sy441*) *Ⅰ* | | | | | | | |
| 5C | EXP.1 | Control | 8.9306±0.407 |  | |  | 144 |
|  | EXP.2 | Control | 10.0964±0.364 |  | |  | 166 |
|  | EXP.3 | Control | 9.2216±0.307 |  | |  | 185 |
|  | EXP.1 | 20℃/200μM HBA | 8.8672±0.331 | # | | -0.71 | 128 |
|  | EXP.2 | 20℃/200μM HBA | 9.9690±0.424 | # | | -1.26 | 129 |
|  | EXP.3 | 20℃/200μM HBA | 9.2065±0.309 | # | | -0.16 | 184 |
| 6B |  |  | RB754/*aak-2(ok524) X* | |  |  |  |
|  | EXP.1 | Control | 13.7582±0.626 |  | |  | 91 |
|  | EXP.2 | Control | 12.6285±0.6 |  | |  | 70 |
|  | EXP.3 | Control | 10.8607±0.239 |  | |  | 79 |
|  | EXP.1 | 20℃/200μM HBA | 13.8500±0.598 | # | | 0.6669 | 80 |
|  | EXP.2 | 20℃/200μM HBA | 12.9866±0.486 | # | | 2.8356 | 75 |
|  | EXP.3 | 20℃/200μM HBA | 10.9000±0.359 | # | | 0.3613 | 70 |
| DA1116/*eat-2*(*ad1116*) *Ⅱ* | | | | | | | |
| 6A | EXP.1 | Control | 22.8021±0.535 |  | |  | 96 |
|  | EXP.2 | Control | 22.7034±0.514 |  | |  | 118 |
|  | EXP.3 | Control | 22.7054±0.47 |  | |  | 112 |
|  | EXP.1 | 20℃/200μM HBA | 26.2908±0.545 | *p*<0.001 | | 15.30 | 141 |
|  | EXP.2 | 20℃/200μM HBA | 26.2578±0.619 | *p*<0.001 | | 15.66 | 128 |
|  | EXP.3 | 20℃/200μM HBA | 26±0.584 | *p*<0.001 | | 14.51 | 139 |
| VC199/*sir-2.1*(*ok434*) *Ⅳ* | | | | | | | |
| 6D | EXP.1 | Control | 20.9268±0.497 |  | |  | 123 |
|  | EXP.2 | Control | 25.0405±0.403 |  | |  | 173 |
|  | EXP.3 | Control | 23.3654±0.563 |  | |  | 104 |
|  | EXP.1 | 20℃/200μM HBA | 20.9573±0.412 | # | | 0.15 | 117 |
|  | EXP.2 | 20℃/200μM HBA | 25±0.367 | # | | -0.16 | 153 |
|  | EXP.3 | 20℃/200μM HBA | 23.5725±0.4 | # | | 0.89 | 138 |
| RB1206/*rsks-1*(*ok1255*) *Ⅲ* | | | | | | | |
| 6C | EXP.1 | Control | 19.4966±0.616 |  | |  | 149 |
|  | EXP.1 | 20℃/200μM HBA | 19.3418±0.496 | # | | -0.79 | 158 |
|  | EXP.2 | Control | 20.1342±0.661 |  | |  | 149 |
|  | EXP.2 | 20℃/200μM HBA | 20.3884±0.551 | # | | 1.26 | 121 |
|  | EXP.3 | Control | 23.3158±0.72 |  | |  | 152 |
|  | EXP.3 | 20℃/200μM HBA | 23.5127±0.62 | # | | 0.84 | 158 |
| CB4876/*clk-1*(*e2519*) *Ⅲ* | | | | | | | |
| 6F | EXP.1 | Control | 23.0093±0.479 |  | |  | 108 |
|  | EXP.1 | 20℃/200μM HBA | 22.7444±0.584 | # | | -1.16 | 90 |
|  | EXP.2 | Control | 22.2222±0.482 |  | |  | 162 |
|  | EXP.2 | 20℃/200μM HBA | 22.5106±0.438 | # | | 1.30 | 141 |
|  | EXP.3 | Control | 22.7730±0.481 |  | |  | 163 |
|  | EXP.3 | 20℃/200μM HBA | 22.2767±0.459 | # | | -2.18 | 159 |
| MQ887/*isp-1*(*qm150*) *Ⅳ* | | | | | | | |
| 6E | EXP.1 | Control | 22.9266±0.3 |  | |  | 177 |
|  | EXP.1 | 20℃/200μM HBA | 23.3880±0.304 | # | | 2.01 | 183 |
|  | EXP.2 | Control | 22.9122±0.319 |  | |  | 148 |
|  | EXP.2 | 20℃/200μM HBA | 23.0329±0.328 | # | | 0.53 | 152 |
|  | EXP.3 | Control | 23.1887±0.324 |  | |  | 159 |
|  | EXP.3 | 20℃/200μM HBA | 23.1549±0.351 | # | | -0.15 | 142 |
| EU1/*skn-1*(*zu67*) *Ⅳ* | | | | | | | |
| 4C | EXP.1 | Control | 11.0928±0.326 |  | |  | 97 |
|  | EXP.1 | 20℃/200μM HBA | 11.1685±0.33 | # | | 0.68 | 89 |
|  | EXP.2 | Control | 10.7551±0.286 |  | |  | 98 |
|  | EXP.2 | 20℃/200μM HBA | 10.8022±0.312 | # | | 0.44 | 91 |
|  | EXP.3 | Control | 10.7195±0.364 |  | |  | 82 |
|  | EXP.3 | 20℃/200μM HBA | 10.6915±0.35 | # | | -0.26 | 94 |

*p*-value was analyzed by log-rank (Mantel-Cox) test.

N: number of dead worms.

*P* < 0.05 indicated that the experiment was statistically significant, while *p* > 0.05 indicated that the experiment was not statistically significant.

**Table S11. Effects of p-hydroxybenzyl alcohol on the mRNA levels of different genes in *C. elegans*.**

| Figure | Strains | Gene | EXP.1 | EXP.2 | EXP.3 | Mean ± SD | *P* value VS *Control* |
| --- | --- | --- | --- | --- | --- | --- | --- |
| 5M | CL4176 | *atg-18* | 2.904 | 2.880 | 2.955 | 2.913 ±0.0386 | *p*<0.001 |
|  |  | *bec-1* | 2.578 | 2.671 | 2.578 | 2.609 ±0.0537 | P=0.001 |
|  |  | *ubl-5* | 1.762 | 1.809 | 1.700 | 1.757 ±0.0546 | *p*<0.001 |
|  |  | *atfs-1* | 1.479 | 1.351 | 1.329 | 1.386 ±0.0807 | P=0.001 |
|  |  | *lgg-1* | 1.100 | 1.190 | 1.252 | 1.181 ±0.0765 | P=0.015 |
|  |  | *dve-1* | 2.250 | 2.241 | 2.249 | 2.247 ±0.0051 | *p*<0.001 |
|  |  | *unc-51* | 2.315 | 2.376 | 2.493 | 2.395 ±0.0904 | *p*<0.001 |
| 1F | CL4176 | *amy-1* | 0.507 | 0.508 | 0.492 | 0.502 ±0.0089 | *p*<0.001 |
| 3I | CF1038 | *sod-3* | 1.057 | 1.135 | 0.890 | 1.027 ±0.1253 | P=0.727 |
|  |  | *ctl-1* | 1.087 | 1.092 | 0.989 | 1.056 ±0.0580 | P=0.170 |
|  |  | *daf-2* | 1.061 | 1.653 | 1.366 | 1.360 ±0.2961 | P=0.103 |
|  |  | *daf-16* | 1.019 | 1.046 | 1.102 | 1.056 ±0.0425 | P=0.085 |
|  |  | *dod-3* | 1.021 | 1.083 | 1.199 | 1.101 ±0.0904 | P=0.125 |
| 4L | EU1 | *skn-1* | 0.850 | 0.960 | 0.802 | 0.871±0.081 | P=0.051 |
|  |  | *gst-4* | 0.792 | 0.934 | 0.951 | 0.892±0.087 | P=0.099 |
|  |  | *gcs-1* | 0.955 | 0.964 | 1.088 | 1.002±0.074 | P=0.957 |
| 3F | N2 | *sod-3* | 1.856 | 1.882 | 1.964 | 1.901±0.057 | *p*<0.001 |
|  |  | *ctl-1* | 1.402 | 1.450 | 1.252 | 1.368 ±0.103 | P=0.004 |
|  |  | *daf-2* | 0.952 | 0.898 | 0.908 | 0.919 ±0.029 | P=0.009 |
|  |  | *daf-16* | 1.671 | 1.740 | 1.761 | 1.724 ±0.047 | *p*<0.001 |
|  |  | *dod-3* | 2.414 | 2.507 | 2.403 | 2.442 ±0.057 | *p*<0.001 |
|  |  | *lgg-1* | 1.765 | 1.744 | 1.724 | 1.744±0.020 | *p*<0.001 |
| 5L |  | *dve-1* | 2.659 | 2.362 | 2.533 | 2.518±0.149 | *p*<0.001 |
|  |  | *atg-18* | 1.293 | 1.174 | 0.954 | 1.141±0.172 | P=0.229 |
|  |  | *atfs-1* | 1.726 | 1.324 | 1.349 | 1.466±0.225 | P=0.023 |
|  |  | *unc-51* | 2.114 | 1.746 | 1.755 | 1.871±0.021 | *p*<0.001 |
|  |  | *ubl-5* | 1.179 | 1.338 | 1.438 | 1.319±0.131 | P=0.013 |
|  |  | *bec-1* | 0.724 | 0.797 | 0.954 | 0.825±0.117 | P=0.062 |
| 5K |  | *hsf-1* | 3.339 | 3.154 | 3.180 | 3.225±0.100 | *p*<0.001 |
|  |  | *hsp-6* | 3.593 | 3.484 | 3.576 | 3.551±0.058 | *p*<0.001 |
|  |  | *hsp-16-1* | 2.928 | 2.949 | 2.764 | 2.881±0.101 | *p*<0.001 |
|  |  | *hsp-16-2* | 3.838 | 3.795 | 3.894 | 3.842±0.050 | *p*<0.001 |
|  |  | *hsp-12-6* | 3.552 | 3.482 | 3.629 | 3.554±0.073 | *p*<0.001 |
|  |  | *hsp-60* | 4.457 | 4.287 | 4.340 | 4.361±0.087 | *p*<0.001 |
| 4F |  | *skn-1* | 3.242 | 3.180 | 3.071 | 3.164±0.087 | *p*<0.001 |
|  |  | *gst-4* | 1.275 | 1.320 | 1.138 | 1.244±0.095 | P=0.011 |
|  |  | *gcs-1* | 2.778 | 2.750 | 2.523 | 2.683±0.140 | *p*<0.001 |
| 5L |  | *ire-1* | 1.161 | 1.144 | 1.148 | 1.151±0.009 | *p*<0.001 |
|  |  | *xbp-1* | 1.542 | 1.450 | 1.444 | 1.479±0.055 | *p*<0.001 |
|  |  | *pek-1* | 1.646 | 1.650 | 1.603 | 1.633±0.026 | *p*<0.001 |

| **Table S12. Primers used for the analysis of gene expression in *C. elegans*.**   \| Gene name \| Primer sequence \| \| --- \| --- \| \| *cdc-42* \| 5'-CTGCTGGACAGGAAGATTACG-3'(F)  5'-CTCGGACATTCTCGAATGAAG-3'(R) \| \| *amy-1* \| 5'-CCG ACA T GACTCAGGATA TGA AGT-3'(F)  5'-CACCATGAGTCCAA TGATTGCA-3'(R) \| \| *daf-16* \| 5'-CCAGACGGAAGGCTTAAAACT-3'(F)  5'-ATTCGCATGAAACGAGAATG-3'(R) \| \| *sod-3* \| 5'-AGCATCATGCCACCTACGTGA-3'(F)  5'-CACCACCATTGAATTTCAGCG-3'(R) \| \| *dod-3* \| 5'-AAGCCATGTTCCCGAATGAG-3'(F)  5'-GCTGCGAAAAGCAAGAAAATG-3'(R) \| \| *ctl-1* \| 5'-GAATGTGAAGAATTATTTCGCTGA-3'(F)  5'-AACTCGATTCCTGGGACGAT-3'(R) \| \| *hsf-1* \| 5'-TTGACGACGACAAGCTTCCAGT-3'(F)  5'-AAAGCTTGCACCAGAATCATCCC-3'(R) \| \| *hsp-6* \| 5'-AGGAACAACAGAGTAAGATTTTC-3'(F)  5'-TCGATTTGGTCCTTGGAAAG-3'(R) \| \| *hsp-16.1* \| 5'-GTCACTTTACCACTATTTCCGTCCAGCTCAACGTTC-3'(F)  5'-CAACGGGCGCTTGCTGAATTGGAATAGATCTTCC-3'(R) \| \| *hsp-16.2* \| 5'-CTGCAGAATCTCTCCATCTGAGTC-3'(F)  5'-AGATTCGAAGCAACTGCACC-3'(R) \| \| *hsp-12.6* \| 5'-GTGATGGCTGACGAAGGAAC-3'(F)  5'-GGGAGGAAGTTATGGGCTTC-3'(R) \| \| *hsp-60* \| 5'-AGGAGAAGCTTAATGAGCG-3'(F)  5'-ACACGGTCCTTCTTCTCT-3'(R) \| \| *dve-1* \| 5'-TCGAGGCCTCATACAAGAA-3'(F)  5'-AAGAGGTTTTCCACAGTGTC-3'(R) \| \| *ubl-5* \| 5'-CAAGAAAAATGATCGAAATCACAGTGAATGATCGTC-3'(F)  5'-GCT CATTGATAATAGAGCTCGAAGTTGAATCCC-3'(R) \| \| *atfs-1* \| 5'-GACGGTCAATTGTGAATTGTC-3'(F)  5'-GCCTAGAAGCTTCCATCATGC-3'(R) \| \| *lgg-1* \| 5'-AACAACTTTGAGAAGCGTCGTGCC-3'(F)  5'-TCTTCTGGACGAAGTTGGATGCGT-3'(R) \| \| *unc-51* \| 5'-TTAGGICATGGAGCATIIGCAA-3'(F)  5'-GGCACGTCTGTGCGATCA-3'(R) \| \| *bec-1* \| 5'-AGGAGCTGGAGCAACAGTTGAAGA-3'(F)  5'-ATATTGACGTTCGGCTTCCAGCGA-3'(R) \| \| *atg-18* \| 5'-TGCAATCTTCCAAACATACGA-3'(F)  5'-CCAAAAATGCCACCAAGCTA-3'(R) \| \| *skn-1* \| 5'-AGTGTCGGCGTTCCAGATTTC-3'(F) \| \|  \| 5'-GTCGACGAATCTTGCGAATCA-3'(R) \| \| *gst-4* \| 5'-TCCGTCAATTCACTTCTTCCG-3'(F) \| \|  \| 5'-AAGAAATCATCACGGGCTGG-3'(R) \| \| *gcs-1* \| 5'-GGAATGCCTTACGGAGGTC-3'(F) \| \|  \| 5'-CGATAGACATGTTTCATCCTTC-3'(R) \| \| *ire-1* \| 5'-TTACATCACGCTTCCCTCGG-3'(F) \| \|  \| 5'-CGAGCACGAACATCATCGGA-3'(R) \| \| *xbp-1* \| 5'-ACGTATTTATGTGCTCCCAG-3'(F) \| \|  \| 5'-TATCATCGCCAAGAAGTTGT-3'(R) \| \| *pek-1* \| 5'-GGGACTAGTCGCAACAGAGC-3'(F) \| \|  \| 5'-GAAGGAAATCCCGCGACTCT-3'(R) \| |
| --- | --- | --- | --- | --- | --- | --- | --- | --- | --- | --- | --- | --- | --- | --- | --- | --- | --- | --- | --- | --- | --- | --- | --- | --- | --- | --- | --- | --- | --- | --- | --- | --- | --- | --- | --- | --- | --- | --- | --- | --- | --- | --- | --- | --- | --- | --- | --- | --- | --- | --- | --- | --- | --- | --- | --- | --- | --- | --- | --- | --- | --- | --- | --- | --- |
|  |
